# The plethora of *Tubeufiaceae* in lakes of the northwestern Yunnan plateau, China

**DOI:** 10.3389/fmicb.2022.1056669

**Published:** 2022-11-28

**Authors:** Long-Li Li, Hong-Wei Shen, Dan-Feng Bao, Dhanushka N. Wanasinghe, Yong-Zhong Lu, Yuan Feng, Zong-Long Luo

**Affiliations:** ^1^College of Agriculture and Biological Science, Dali University, Dali, China; ^2^Center of Excellence in Fungal Research, Mae Fah Luang University, Chiang Rai, Thailand; ^3^School of Science, Mae Fah Luang University, Chiang Rai, Thailand; ^4^Department of Entomology and Plant Pathology, Faculty of Agriculture, Chiang Mai University, Chiang Mai, Thailand; ^5^Centre for Mountain Futures, Kunming Institute of Botany, Chinese Academy of Sciences, Honghe, China; ^6^School of Food and Pharmaceutical Engineering, Guizhou Institute of Technology, Guiyang, China

**Keywords:** *Dothideomycetes*, lignicolous freshwater fungi, helicosporous hyphomycetes, morphology, multigene phylogeny

## Abstract

The diversity of lignicolous freshwater fungi in northwestern Yunnan, China, has been studied for several years in the College of Agriculture and Biological Science, at Dali University. Over the last 5 years, we published two new genera and nine new species of *Tubeufiaceae* from northwestern Yunnan. This study focused on introducing tubeufia-like hyphomycetous fungi found in freshwater lakes in the northwestern Yunnan plateau. Eleven fresh collections of tubeufiaceous taxa were gathered and identified. Among them, a new genus, *Neomanoharachariella*, is introduced to accommodate *Neomanoharachariella aquatica*, which is characterized by a light brown to dark brown color, dictyoseptate, and broadly oval to ellipsoid and well-developed conidiophores. Two new species, *viz*., *Neohelicosporium suae* and *Parahelicomyces suae*, one new record, *Helicoma rufum*, and three new collections, namely, *H. rugosum, P. hyalosporus*, and *Tubeufia cylindrothecia* are introduced based on morphological evidence and molecular phylogenetic analysis of combined ITS, LSU, *tef 1-*α, and RPB2 sequence data. Detailed descriptions and illustrations of these species are provided, and a morphological comparison with similar taxa is discussed.

## Introduction

Lignicolous freshwater fungi are an important group of organisms, involved in nutrient cycling by decaying submerged wood (Hyde et al., 2016a; Shen et al., 2022). Yunnan Province is one of the richest biodiversity hotspots, containing abundant resources of lignicolous freshwater fungi, with more than 281 species reported since 1986 (Shen et al., 2022). Among lignicolous freshwater fungi, *Tubeufiales* is one of the most species-rich groups in *Dothideomycetes*. *Tubeufiales* was introduced by Boonmee et al. (2014) based on molecular phylogenetic analysis to accommodate *Tubeufiaceae*. Liu et al. (2017) treated *Bezerromycetaceae* and *Wiesneriomycetaceae* as accepted families in *Tubeufiales* based on divergence time estimates. To date, *Tubeufiales* contains three families, *viz*., *Bezerromycetaceae, Tubeufiaceae*, and *Wiesneriomycetaceae*. The majority of *Tubeufiaceae* comprised freshwater taxa (Doilom et al., 2017; Lu et al., 2018a,b; Dong et al., 2020; Hongsanan et al., 2020). The family was established by Barr (1979) based on the generic type *Tubeufia* (Penzig and Saccardo, 1897). In the last decade, several studies of *Tubeufiaceae* have been published, with many species reported in freshwater habitats; most of them were asexual morphs (Boonmee et al., 2011; Hyde et al., 2016b, 2017; Brahmanage et al., 2017; Luo et al., 2017; Liu et al., 2018; Lu et al., 2018a,b). Lu et al. (2018b) reappraised and provided an updated phylogenetic tree for *Tubeufiales* which included 13 new genera, and expanded the circumscription of the type family *Tubeufiaceae*. To date, *Tubeufiaceae* includes 47 genera. They are widely distributed in tropical, subtropical, and temperate regions (Boonmee et al., 2011, 2014; Luo et al., 2017; Lu et al., 2018b), and most taxa are saprobic on woody substrates in terrestrial and freshwater habitats (Cai et al., 2003; Zhao et al., 2007; Lu et al., 2018b).

Members of *Tubeufiaceae* are a group of microfungi that are morphologically fascinating (Zhao et al., 2007) and have helicosporous hyphomycetes. *Tubeufiaceae* has been reported as sexual and asexual morphs. Asexual morphologies are mostly found as helicosporous hyphomycetes, while some are phragmosporous and chlamydosporous conidia (Lu et al., 2018b; Dong et al., 2020). Helicosporous hyphomycetes make up a large part of the order *Tubeufiales*. They are known to be present in many genera, such as *Acanthohelicosporium, Berkleasmium, Chlamydotubeufia, Dematiohelicosporum, Helicangiospora, Helicodochium, Helicohyalinum, Helicoma, Helicomyces, Helicosporium, Helicotubeufia, Neoacanthostigma, Neohelicomyces, Neohelicosporium, Parahelicomyces*, and *Tubeufia* (Boonmee et al., 2011, 2014; Brahmanage et al., 2017; Lu et al., 2017a,b,c, 2018a,b; Luo et al., 2017; Liu et al., 2018). Chlamydosporous and phragmosporous hyphomycetes in *Tubeufiaceae* are reported in *Aquaphila, Berkleasmium, Chlamydotubeufia, Dictyospora, Helicoma, Kamalomyces, Neochlamydotubeufia, Tamhinispora*, and *Tubeufia* (Lu et al., 2018b). Their sexual morphs are characterized by superficial ascomata, bitunicate asci, and hyaline to pale brown, elongate, obovoid or oblong, and septate ascospores (Barr, 1980; Kodsueb et al., 2006; Boonmee et al., 2011, 2014; Brahmanage et al., 2017; Lu et al., 2018b).

*Helicoma* was introduced by Corda (1837) with the type species *H. muelleri*. It is one of the earliest described helicosporous genus (Morgan, 1892; Linder, 1929; Moore, 1955). *Helicoma* includes two asexual morphs, one is characterized by conidia pleurogenous, helicoid, becoming loosely coiled in water, conidiogenous cells with denticles, and tooth-like protrusions. Other conidia are acrogenous, helicoid, circinate, tapering toward the apex, truncating at the base, and not becoming loose in water (Lu et al., 2018b). *Neohelicosporium* was introduced by Lu et al. (2018a) based on phylogenetic and morphological evidence. Currently, 24 species are accepted in the genus, of which 11 species were reported in freshwater habitats. *Pseudohelicomyces* was established by Lu et al. (2018b) to accommodate five species, *viz*., *Ps. aquaticus, Ps. hyalosporus, Ps. indicus, Ps. paludosus*, and *Ps. talbotii* (type species) based on multi-gene phylogenetic analysis. However, following previous publications, this generic name has an older homonym: *Pseudohelicomyces* (Valenzuela and Garnica, 2000), and this rendered the *Pseudohelicomyces* described by Lu et al. illegitimate. Lu et al. (2020) provided a proposal to conserve *Pseudohelicomyces* (*Tubeufiaceae*) against *Pseudohelicomyces* (*Hymenogastraceae*). Hsieh et al. (2021) established *Parahelicomyces* to replace *Pseudohelicomyces* and transferred all species of *Pseudohelicomyces* to *Parahelicomyces*. Until recently, nine species are accepted in *Parahelicomyces* (Lu et al., 2018b; Li et al., 2022; Tian et al., 2022). *Tubeufia* is the largest genus in *Tubeufiaceae* and is commonly reported as saprobes on submerged decaying wood in freshwater habitats (Ho et al., 2001; Cai et al., 2002; Liu et al., 2018; Lu et al., 2018b; Jayasiri et al., 2019). Members of *Tubeufiaceae* are mostly saprobic and widely distributed and are often found on woody substrates in terrestrial and freshwater habitats (Lu et al., 2018b). The southern China areas of Guangdong, Guangxi, Guizhou, Hubei, Yunnan, and other subtropical or tropical regions are very suitable for the growth and distribution of *Tubeufiaceae* fungi (Cai et al., 2002; Liu et al., 2018; Lu et al., 2018a,b).

During our investigation of freshwater fungi on submerged decaying wood, more than 100 specimens of freshwater hyphomycetes were collected from the lakes in the northwestern Yunnan plateau. This article aims to introduce eleven helicosporous hyphomycetes which were collected from the Luguhu and Shuduhu lakes. Phylogenetic analyses of combined ITS, LSU, *tef 1-*α, and RPB2 sequence data place them in *Helicoma, Neohelicosporium, Parahelicomyces*, and *Tubeufia*. A new genus *Neomanoharachariella* and three new species, *viz*., *Neomanoharachariella aquatica, Neohelicosporium suae*, and *Parahelicomyces suae* are introduced with morphological and phylogenetic evidence. *Helicoma rufum* is newly recorded in freshwater habitats for the first time in China. In addition, we combine *Helicoma* sp. (HKUCC 9118) as *H. rugosum* (HKUCC 9118) according to multi-gene phylogeny analysis and morphological evidence. Three known species, namely, *Helicoma rugosum, Parahelicomyces hyalosporus*, and *Tubeufia cylindrothecia*, are also accounted. Full descriptions, color photo plates of the species, and an updated phylogenetic tree for *Tubeufiaceae* are provided. This study provides a case study for lakes as a worthwhile niche area for the further study of hyphomycetous associations and hints that these lakes in the Yunnan plateau may potentially host numerous unknown fungal species.

## Materials and methods

### Collection, isolation, and morphology

Specimens of submerged decaying wood were collected from the Luguhu and Shuduhu lakes in the northwestern Yunnan province of China and were taken to the laboratory in ziplock plastic bags. The specimens were incubated at room temperature for 1 week in plastic boxes lined with moistened tissue paper. Specimen observations and isolation were performed by following the protocols provided by Luo et al. (2018) and Senanayake et al. (2020). Macromorphological characteristics of samples were observed using an Optec SZ 760 compound stereomicroscope. Temporarily prepared microscope slides were placed under a Nikon ECLIPSE Ni-U compound stereomicroscope for observation and micro-morphological-photography. The morphologies of colonies on native substrates were photographed with a Nikon SMZ1000 stereo zoom microscope. Single spore isolation was performed according to the following steps: the conidia suspension from specimens was transported using a sterilized pipette, placed on potato dextrose agar (PDA), and incubated at room temperature overnight. Germinated conidia were transferred to new PDA/malt extract agar (MEA) (Beijing land bridge technology CO., LTD., China) plates and incubated at room temperature (25°C). The specimens were deposited in the Herbarium of Cryptogams Kunming Institute of Botany, Academia Sinica (KUN-HKAS), Kunming, China. Living cultures were deposited in the China General Microbiological Culture Collection Center (CGMCC), Beijing, China, and the Kunming Institute of Botany Culture Collection Center, Kunming, China (KUNCC). Mycobank numbers were registered (https://www.mycobank.org). New species were established following the recommendations outlined by Chethana et al. (2021).

### DNA extraction, PCR amplification, and sequencing

Fungal mycelium was removed from the surfaces of colonies that were grown on PDA or MEA for 4–6 weeks and transferred to a 1.5 ml centrifuge tube. A Trelief TM Plant Genomic DNA Kit (TSP101-50) was used to extract DNA from the ground mycelium according to the manufacturer's instructions. Four gene regions; ITS, LSU, *tef 1-*α, and RPB2 were amplified using ITS5/ITS4, LR0R/LR5 (Vilgalys and Hester, 1990), 983F/2218R, and fRPB2-5F/fRPB2-7cR (Liu et al., 1999). The PCR mixture was prepared as follows: 12.5 μl of 2 × Taq Master Mix (Genes and Biotech Co., Ltd), 1 μl of each primer, 1 μl of genomic DNA extract, and 9.5 μl of deionized water. The PCRs of ITS, LSU, *tef 1-*α, and RPB2 genes were processed as described in Su et al. (2015). PCR amplification was confirmed on 1% agarose electrophoresis gels stained with ethidium bromide. Sequencing was carried out by Tsingke Biological Engineering Technology and Services Co., Ltd (Yunnan, P.R. China).

### Sequence alignment

Sequences were assembled using BioEdit. A BLAST search was performed on sequences with high similarity indices to find the closest matches with taxa in *Tubeufiaceae* and in recently published data (Luo et al., 2017; Lu et al., 2018b; Dong et al., 2020). All consensus sequences and the reference sequences were automatically aligned with MAFFT version 7.0 (Kuraku et al., 2013; Katoh et al., 2019). Aligned sequences of each gene region (ITS, LSU, *tef 1-*α, and RPB2) were combined and manually improved using BioEdit v. 7.0 (Hall, 1999). Ambiguous regions were excluded from the analysis and gaps were treated as missing data.

### Phylogenetic analyses

Phylogenetic analyses were performed using maximum likelihood (ML) and Bayesian tree building criteria. Maximum likelihood (ML) analysis was carried out using RAxML-HPC2 on XSEDE (8.2.12) (Stamatakis, 2006; Stamatakis et al., 2008) on the CIPRES Science Gateway website (Miller et al., 2010: http://www.phylo.org/portal2) and the estimated proportion of invariant sites was determined using the GTRGAMMA+I model. Bayesian analyses were performed using MrBayes v. 3.1.2. (Ronquist and Huelsenbeck, 2003). The model of each gene was estimated using MrModeltest 2.3, and the GTR + I + G model was the best-fit model for ITS, LSU, *tef 1-*α, and RPB2 Bayesian analyses. Posterior probabilities (PP) (Ranala and Yang, 1996) were performed by Markov chain Monte Carlo sampling (BMCMC) in MrBayes v.3.1.2 (Liu et al., 2012). Six simultaneous Markov chains were run for 10 million generations, and trees were sampled every 100th generation (resulting in 100,000 trees). The first 20,000 trees, representing the burn-in phase of the analyses, were discarded and the remaining 80,000 (post-burning) trees were used for calculating PP in the majority rule consensus tree (Cai et al., 2006; Liu et al., 2012). Phylogenetic trees were represented by FigTree v. 1.4.0 and edited in Microsoft Office PowerPoint 2016. Newly-generated sequences in this study were submitted to GenBank, and the strain information used in this paper is provided in Table 1.

**Table 1:** GenBank numbers and culture collection accession numbers of species included in the phylogenetic study.

**Taxa**	**Strain**	**GenBank Accession No**.			
		**ITS**	**LSU**	***tef 1-α***	**RPB2**
*Acanthohelicosporapinicola* ^T^	MFLUCC 10–0116	KF301526	KF301534	KF301555	–
*Acanthohelicospora scopula*	ANM 386	GQ856141	GQ850489	–	–
*Acanthostigmina multiseptatum*	ANM 475	GQ856145	GQ850492	–	–
*Acanthostigmina multiseptatum*	ANM 665	GQ856144	GQ850493	–	–
*Acanthotubeufiafiliforme* ^T^	ANM 101	–	GQ850495	–	–
*Acanthotubeufia filiforme*	ANM 514	GQ856146	GQ850494	–	–
*Acanthotubeufia albicans*	BCC 3463	DQ341097	DQ341100	–	–
*Acanthotubeufia albicans*	BCC 3520	DQ341098	DQ341102	–	–
*Acanthotubeufia albicans*	BCC 3543	DQ341096	DQ341101	–	–
*Acanthotubeufia albicans*	MFLUCC 16–0010	KX454165	KX454166	KY117034	MF535255
*Acanthotubeufia albicans*	MFLUCC 16–0020	KX454167	KX454168	–	MF535256
*Berkleasmiumaquaticum* ^T^	MFLUCC 17–0049	KY790444	KY790432	KY792608	MF535268
*Berkleasmiumfusiforme* ^T^	MFLUCC 17–1978	MH558693	MH558820	MH550884	MH551007
*Berkleasmiumguangxiense* ^T^	MFLUCC 17–0042	KY790448	KY790436	KY792612	MF535270
*Berkleasmiumlongisporum* ^T^	MFLUCC 17–1999	MH558698	MH558825	MH550889	MH551012
*Boerlagiomycesmacrospora* ^T^	MFLUCC 12–0388	KU144927	KU764712	KU872750	–
*Botryosphaeria dothidea*	CBS 115476	KF766151	DQ678051	DQ767637	DQ677944
*Chlamydotubeufiacylindrica* ^T^	MFLUCC 16–1130	MH558702	MH558830	MH550893	MH551018
*Chlamydotubeufiahuaikangplaensis* ^T^	MFLUCC 10–0926	JN865210	JN865198	–	–
*Chlamydotubeufiakrabiensis* ^T^	MFLUCC 16–1134	KY678767	KY678759	KY792598	MF535261
*Dematiohelicoma pulchrum*	MUCL 39827	AY916457	AY856872	–	–
*Dematiohelicomyceshelicosporus* ^T^	MFLUCC 16–0213	KX454169	KX454170	KY117035	MF535258
*Dematiohelicomyces helicosporus*	MFLUCC 16–0003	MH558703	MH558831	MH550894	MH551019
*Dematiohelicomyces helicosporus*	MFLUCC 16–0007	MH558704	MH558832	MH550895	MH551020
*Dematiohelicosporumguttulatum* ^T^	MFLUCC 17–2011	MH558705	MH558833	MH550896	MH551021
*Dematiotubeufiachiangraiensis* ^T^	MFLUCC 10–0115	JN865200	JN865188	KF301551	–
*Dictyosporathailandica* ^T^	MFLUCC 16–0001	KY873627	KY873622	KY873286	MH551023
*Dictyospora thailandica*	MFLUCC 11–0512	KF301528	KF301536	–	–
*Dictyospora thailandica*	MFLUCC 16–0215	KY873628	KY873623	KY873287	–
*Helicangiosporalignicola* ^T^	MFLUCC 11–0378	KF301523	KF301531	KF301552	–
*Helicoarctatusaquaticus* ^T^	MFLUCC 17–1996	MH558707	MH558835	MH550898	MH551024
*Helicoarctatusthailandicus* ^T^	MFLUCC 18–0332	–	ON764311	MK541685	–
*Helicodochium aquaticum*	MFLUCC 16–0008	MH558708	MH558836	MH550899	MH551025
*Helicodochiumaquaticum* ^T^	MFLUCC 17–2016	MH558709	MH558837	MH550900	MH551026
*Helicohyalinum aquaticum*	MFLUCC 16–1131	KY873625	KY873620	KY873284	MF535257
*Helicohyalinuminfundibulum* ^T^	MFLUCC 16–1133	MH558712	MH558840	MH550903	MH551029
*Helicoma ambiens*	UAMH 10533	AY916451	AY856916	–	–
*Helicoma ambiens*	UAMH 10534	AY916450	AY856869	–	–
*Helicomaaquaticum* ^T^	MFLUCC 17–2025	MH558713	MH558841	MH550904	MH551030
*Helicomabrunneisporum* ^T^	MFLUCC 17–1983	MH558714	MH558842	MH550905	MH551031
*Helicoma dennisii*	NBRC 30667	AY916455	AY856897	–	–
*Helicoma fusiforme* ^T^	MFLUCC 17–1981	MH558715	–	MH550906	–
*Helicomaguttulatum* ^T^	MFLUCC 16–0022	KX454171	KX454172	MF535254	MH551032
*Helicoma hongkongense*	MFLUCC 17–2005	MH558716	MH558843	MH550907	MH551033
*Helicomainthanonense* ^T^	MFLUCC 11–0003	JN865211	JN865199	–	–
*Helicomakhunkornensis* ^T^	MFLUCC 10–0119	JN865203	JN865191	KF301559	–
*Helicoma linderi*	NBRC 9207	AY916454	AY856895	–	–
*Helicoma longisporum*	MFLUCC 16–0002	MH558717	MH558844	MH550908	MH551034
*Helicoma longisporum*	MFLUCC 16–0005	MH558718	–	MH550909	MH551035
*Helicoma longisporum*	MFLUCC 16–0211	MH558719	MH558845	MH550910	MH551036
*Helicomalongisporum* ^T^	MFLUCC 17–1997	MH558720	MH558846	MH550911	MH551037
*Helicomamiscanthi* ^T^	MFLUCC 11–0375	KF301525	KF301533	KF301554	–
*Helicoma muelleri*	CBS 964.69	AY916453	AY856877	–	–
*Helicoma muelleri*	UBC F13877	AY916452	AY856917	–	–
*Helicomamultiseptatum* ^T^	GZCC 16–0080	MH558721	MH558847	MH550912	MH551038
*Helicoma nematosporum*	MFLUCC 16–0011	MH558722	MH558848	MH550913	MH551039
*Helicomarubriappendiculatum* ^T^	MFLUCC 18–0491	MH558723	MH558849	MH550914	MH551040
*Helicomarufum* ^T^	MFLUCC 17–1806	MH558724	MH558850	MH550915	–
***Helicoma rufum***	**CGMCC 3.23543**	**OP184080**	**OP184069**	**OP186053**	**OP186061**
*Helicoma rugosum*	ANM 196	GQ856138	GQ850482	–	–
*Helicoma rugosum*	ANM 953	GQ856139	GQ850483	–	–
*Helicoma rugosum*	ANM 1169	–	GQ850484	–	–
*Helicoma rugosum*	JCM 2739	–	AY856888	–	–
***Helicoma rugosum***	**KUNCC 22–12445**	**OP184078**	**OP184067**	**OP186051**	–
*Helicoma rugosum*	HKUCC 9118	–	AY849966	–	–
*Helicoma septoconstrictum*	MFLUCC 17–1991	MH558725	MH558851	MH550916	MH551041
*Helicomaseptoconstrictum* ^T^	MFLUCC 17–2001	MH558726	MH558852	MH550917	MH551042
*Helicomasiamense* ^T^	MFLUCC 10–0120	JN865204	JN865192	KF301558	–
*Helicomatectonae* ^T^	MFLUCC 12–0563	KU144928	KU764713	KU872751	–
*Helicoma vaccinii*	CBS 216.90	AY916486	AY856879	–	–
*Helicomyces hyalosporus*	GZCC 16–0070	MH558728	MH558854	MH550919	MH551044
*Helicomyceshyalosporus* ^T^	MFLUCC 17–0051	MH558731	MH558857	MH550922	MH551047
*Helicomyces torquatus*	MFLUCC 16–0217	MH558732	MH558858	MH550923	MH551048
*Helicomyceschiayiensis* ^T^	BCRC FU30842	LC316604	–	–	–
*Helicomyces colligatus*	MFLUCC 16–1132	MH558727	MH558853	MH550918	MH551043
*Helicosporiumflavum* ^T^	MFLUCC 16–1230	KY873626	KY873621	KY873285	–
*Helicosporiumluteosporum* ^T^	MFLUCC 16–0226	KY321324	KY321327	KY792601	MH551056
*Helicosporiumvesicarium* ^T^	MFLUCC 17–1795	MH558739	MH558864	MH550930	MH551055
*Helicotruncatum palmigenum*	NBRC 32663	AY916480	AY856898	–	–
*Helicotubeufiaguangxiensis* ^T^	MFLUCC 17–0040	MH290018	MH290023	MH290028	MH290033
*Helicotubeufiahydei* ^T^	MFLUCC 17–1980	MH290021	MH290026	MH290031	MH290036
*Helicotubeufiajonesii* ^T^	MFLUCC 17–0043	MH290020	MH290025	MH290030	MH290035
*Kamalomyces thailandicus*	MFLUCC 11–0158	MF506883	MF506881	MF506885	–
*Kamalomycesthailandicus* ^T^	MFLUCC 13–0233	MF506884	MF506882	MF506886	–
*Manoharachariellatectonae* ^T^	MFLUCC 12–0170	KU144935	KU764705	KU872762	–
*Muripulchra aquatica*	DLUCC 0571	KY320531	KY320548	–	–
*Muripulchra aquatica*	KUMCC 15–0245	KY320533	KY320550	KY320563	MH551057
*Muripulchra aquatica*	KUMCC 15–0276	KY320534	KY320551	KY320564	MH551058
*Muripulchraaquatica* ^T^	MFLUCC 15–0249	KY320532	KY320549	–	–
*Neoacanthostigmafusiforme* ^T^	MFLUCC 11–0510	KF301529	KF301537	–	–
*N* *eochlamydotubeufiafusiformis* ^T^	MFLUCC 16–0016	MH558740	MH558865	MH550931	MH551059
*Neochlamydotubeufia fusiformis*	MFLUCC 16–0214	MH558741	MH558866	MH550932	MH551060
*Neochlamydotubeufiakhunkornensis* ^T^	MFLUCC 10–0118	JN865202	JN865190	KF301564	–
*Neochlamydotubeufia khunkornensis*	MFLUCC 16–0025	MH558742	MH558867	MH550933	MH551061
*Neohelicoma fagacearum*	MFLUCC 11–0379	KF301524	KF301532	KF301553	–
*Neohelicomycesaquaticus* ^T^	MFLUCC 16–0993	KY320528	KY320545	KY320561	MH551066
*Neohelicomycesgrandisporus* ^T^	KUMCC 15–0470	KX454173	KX454174	–	MH551067
*Neohelicomycessubmersus* ^T^	MFLUCC 16–1106	KY320530	KY320547	–	MH551068
*Neohelicosporium abuense*	CBS 101688	AY916470	AY916085	–	–
*Neohelicosporiumacrogenisporum* ^T^	MFLUCC 17–2019	MH558746	MH558871	MH550937	MH551069
*Neohelicosporiumaquaticum* ^T^	MFLUCC 17–1519	MF467916	MF467929	MF535242	MF535272
*Neohelicosporiumastrictum* ^T^	MFLUCC 17–2004	NR_160377	NG_068566	MH550938	MH551070
*Neohelicosporium aurantiellum*	ANM 718	GQ856140	GQ850485	–	–
*Neohelicosporiumbambusicola* ^T^	MFLUCC 21–0156	OL606157	OL606146	OL964517	OL964523
*Neohelicosporiumellipsoideum* ^T^	MFLUCC 16–0229	MH558748	MH558873	MH550939	MH551071
*Neohelicosporiumfusisporum* ^T^	MFUCC 16–0642	MG017612	MG017613	MG017614	–
*Neohelicosporium griseum*	UAMH 1694	AY916473	AY856902	–	–
*Neohelicosporium guangxiense*	GZCC 16–0068	MH558749	MH558874	MH550940	MH551072
*Neohelicosporiumguangxiense* ^T^	MFLUCC 17–1522	MF467922	MF467935	MF535248	MF535278
*Neohelicosporiumhyalosporum* ^T^	GZCC 16–0076	MF467923	MF467936	MF535249	MF535279
*Neohelicosporiumirregulare* ^T^	MFLUCC 17–1796	MH55875	MH558877	MH550943	MH551075
*Neohelicosporium krabiense*	MFLUCC 16–0224	MH558754	MH558879	MH550945	MH551077
*Neohelicosporiumlaxisporum* ^T^	MFLUCC 17–2027	MH558755	MH558880	MH550946	MH551078
*Neohelicosporium morganii*	CBS 281.54	MH857331	MH868874	–	–
*Neohelicosporium morganii*	CBS 222.58	AY916469	AY856880	–	–
*Neohelicosporiumovoideum* ^T^	GZCC 16–0064	MH558756	MH558881	MH550947	MH551079
*Neohelicosporium panacheum*	CBS 257.59	MH857857	–	–	–
*Neohelicosporium parvisporum*	GZCC 16–0078	MF467924	MF467937	MF535250	MF535280
*Neohelicosporium parvisporum*	MFLUCC 17–1523	MF467926	MF467939	MF535252	MF535282
*Neohelicosporium* sp.	HKUCC 10235	–	AY849942	–	–
*Neohelicosporium* sp.	CBS 189.95	AY916472	AY856882	–	–
*Neohelicosporium submersum*	MFLUCC 17–2376	NR_171979	MN913738	–	–
***Neohelicosporiumsuae*** ^**T**^	**CGMCC 3.23541**	**OP184079**	**OP184068**	**OP186052**	**OP265702**
*Neohelicosporiumtaiwanense* ^T^	BCRC FU30841	LC316603	–	–	–
*Neohelicosporiumthailandicum* ^T^	MFLUCC 16–0221	MF467928	MF467941	MF535253	MF535283
***Neomanoharachariellaaquatica*** ^**T**^	**CGMCC 3.23539**	**OP184074**	**OP184063**	**OP186047**	**OP186058**
***Neomanoharachariella aquatica***	**CGMCC 3.23540**	**OP184075**	**OP184064**	**OP186048**	**OP186059**
*Neotubeufiakrabiensis* ^T^	MFLUCC 16–1125	MG012031	MG012024	MG012010	MG012017
*Parahelicomycesaquaticus* ^T^	MFLUCC 16–0234	MH558766	MH558891	MH550958	MH551092
*Parahelicomyceschiangmaiensis* ^T^	MFLUCC 21–0159	OL697884	OL606145	OL964516	OL964522
*Parahelicomyces hyalosporus*	CBS 283.51	AY916464	AY856881	DQ677928	DQ677981
*Parahelicomyces hyalosporus*	KUMCC 15–0281	KY320526	KY320543	KY320559	MH551089
*Parahelicomyces hyalosporus*	KUMCC 15–0322	KY320525	KY320542	KY320558	–
*Parahelicomyces hyalosporus*	KUMCC 15–0411	KY320527	KY320544	KY320560	–
*Parahelicomyces hyalosporus*	KUMCC 15–0430	KY320524	KY320541	KY320557	MH551090
*Parahelicomyceshyalosporus* ^T^	MFLUCC 15–0343	KY320523	KY320540	–	–
***Parahelicomyces hyalosporus***	**CGMCC 3.23535**	**OP184073**	**OP184062**	**OP186046**	**OP186057**
***Parahelicomyces hyalosporus***	**KUNCC 22–12443**	**OP184076**	**OP184065**	**OP186049**	–
***Parahelicomyces hyalosporus***	**KUNCC 22–12444**	**OP184077**	**OP184066**	**OP186050**	**OP186060**
*Parahelicomyces indicus*	CBS 374.93	AY916477	AY856885	–	–
*Parahelicomycesmenglunicus* ^T^	KUN HKAS 85795	MK335914	–	MK335916	–
*Parahelicomyces paludosus*	CBS 120503	DQ341095	DQ341103	–	–
*Parahelicomyces quercus*	MFUCC 17–0895	MK347720	MK347934	MK360077	MK434906
***Parahelicomycessuae*** ^**T**^	**CGMCC 3.23534**	**OP184072**	**OP184061**	**OP186045**	**OP186056**
***Parahelicomyces suae***	**CGMCC 3.23538**	**OP184081**	**OP184070**	**OP186054**	–
*Parahelicomyces talbotii*	MUCL 33010	AY916465	AY856874	–	–
*Parahelicomyces talbotii* ^**T**^	MFLUCC 17–2021	MH558765	MH558890	MH550957	MH551091
*Parahelicomycesyunnanensis* ^T^	CGMCC 3.20429	MZ092717	MZ841658	–	OM022000
*Pleurohelicosporiumparvisporum* ^T^	MFLUCC 17–1982	MH558764	MH558889	MH550956	MH551088
*Pseudohelicoon gigantisporum*	BCC 3550	AY916467	AY856904	–	–
*Pseudohelicoonsubglobosum* ^T^	BCRC FU30843	LC316607	LC316610	–	–
*Tamhinispora indica*	NFCCI 2924	KC469282	KC469283	–	–
*Tamhinispora srinivasanii*	NFCCI 4231	MG763746	MG763745	–	–
*Thaxteriellopsis lignicola*	MFLUCC 10–0123	JN865207	JN865195	KF301562	–
*Thaxteriellopsis lignicola*	MFLUCC 10–0124	JN865208	JN865196	KF301561	–
*Tubeufiaabundata* ^T^	MFLUCC 17–2024	MH558769	MH558894	MH550961	MH551095
*Tubeufia amazonensis*	ATCC 42524	AY916458	AY856911	–	–
*Tubeufiaaquatica* ^T^	MFLUCC 16–1249	KY320522	KY320539	KY320556	MH551142
*Tubeufia aquatica*	MFLUCC 17–1794	MH558770	MH558895	MH550962	MH551096
*Tubeufiabambusicola* ^T^	MFLUCC 17–1803	MH558771	MH558896	MH550963	MH551097
*Tubeufiabrevis* ^T^	MFLUCC 17–1799	MH558772	MH558897	MH550964	MH551098
*Tubeufiabrunnea* ^T^	MFLUCC 17–2022	MH558773	MH558898	MH550965	MH551099
*Tubeufiachiangmaiensis* ^T^	MFLUCC 11–0514	KF301530	KF301538	KF301557	–
*Tubeufia chiangmaiensis*	MFLUCC 17–1801	MH558774	MH558899	MH550966	MH551100
*Tubeufiachlamydospora* ^T^	MFLUCC 16–0223	MH558775	MH558900	MH550967	MH551101
*Tubeufiacocois* ^T^	MFLUCC 22–0001	OM102541	OL985957	OM355486	OM355491
*Tubeufia cylindrothecia*	MFLUCC 16–1253	KY320519	KY320536	KY320553	–
*Tubeufia cylindrothecia*	MFLUCC 16–1283	KY320518	KY320535	KY320552	MH551143
*Tubeufia cylindrothecia*	MFLUCC 17–1792	MH558776	MH558901	MH550968	MH551102
*Tubeufia cylindrothecia*	MFLUCC 11–0076	MT627709	MN913702	–	–
*Tubeufia cylindrothecia*	MFLUCC 10–0919	MT627710	MN913701	–	–
***Tubeufia cylindrothecia***	**CGMCC 3.23552**	**OP184071**	**OP184060**	**OP186044**	**OP186055**
*Tubeufiadictyospora* ^T^	MFLUCC 17–1805	MH558778	MH558903	MH550970	MH551104
*Tubeufia eccentrica*	GZCC 16–0084	MH558781	MH558906	MH550973	MH551107
*Tubeufiaeccentrica* ^T^	MFLUCC 17–1524	MH558782	MH558907	MH550974	MH551108
*Tubeufia entadae*	MFLU 18–2102	NR163323	–	–	–
*Tubeufiafangchengensis* ^T^	MFLUCC 17–0047	MH558783	MH558908	MH550975	MH551109
*Tubeufiafiliformis* ^T^	MFLUCC 16–1128	–	KY092407	KY117028	MF535284
*Tubeufia filiformis*	MFLUCC 16–1135	KY092416	KY092411	KY117032	MF535285
*Tubeufiageniculata* ^T^	BCRC FU30849	LC335817	–	–	–
*Tubeufia geniculata*	NCYU U2–1B	LC335816	–	–	–
*Tubeufia guangxiensis*	MFLUCC 17–0045	MG012025	MG012018	–	–
*Tubeufiahechiensis* ^T^	MFLUCC 17–0052	MH558785	MH558910	MH550978	MH551112
*Tubeufiahyalospora* ^T^	MFLUCC 15–1250	MH558786	MH558911	MH550979	–
*Tubeufia inaequalis*	MFLUCC 17–0053	MH558789	MH558914	MH550982	MH551115
*Tubeufia inaequalis*	MFLUCC 17–1998	MH558791	MH558916	MH550984	MH551117
*Tubeufia javanica*	MFLUCC 12–0545	KJ880034	KJ880036	–	–
*Tubeufiakrabiensis* ^T^	MFLUCC 16–0228	MH558792	MH558917	MH550985	MH551118
*Tubeufialatispora* ^T^	MFLUCC 16–0027	KY092417	KY092412	KY117033	MH551119
*Tubeufia laxispora*	MFLUCC 16–0219	KY092414	KY092409	KY117030	MF535286
*Tubeufialaxispora* ^T^	MFLUCC 16–0232	KY092413	KY092408	KY117029	MF535287
*Tubeufia laxispora*	MFLUCC 17–2023	MH558794	MH558919	MH550987	MH551121
*Tubeufia lilliputea*	NBRC 32664	AY916483	AY856899	–	–
*Tubeufialongihelicospora* ^T^	MFLUCC 16–0753	MZ538531	MZ538565	MZ567106	–
*Tubeufia longihelicospora*	MFLUCC 21–0151	OL606156	OL606149	OL964520	OL964526
*Tubeufialongiseta* ^T^	MFLUCC 15–0188	KU940133	–	–	–
*Tubeufia machaerinae*	MFLUCC 17–0055	MH558795	MH558920	MH550988	MH551122
*Tubeufiamackenziei* ^T^	MFLUCC 16–0222	KY092415	KY092410	KY117031	MF535288
*Tubeufianigroseptum* ^T^	CGMCC 3.20430	MZ092716	MZ853187	OM022002	OM022001
*Tubeufia parvispora*	MFLUCC 17–1992	MH558796	MH558921	MH550989	MH551123
*Tubeufia parvispora*	MFLUCC 17–2009	MH558798	MH558923	MH550991	MH551125
*Tubeufiaroseohelicospora* ^T^	MFLUCC 15–1247	KX454177	KX454178	–	MH551144
*Tubeufiarubra* ^T^	GZCC 16–0081	MH558801	MH558926	MH550994	MH551128
*Tubeufiasahyadriensis* ^T^	NFCCI 4252	MH033849	MH033850	MH033851	–
*Tubeufia sahyadriensis*	RAJ 99.2	MN393081	MN393082	MN393083	–
*Tubeufia sessilis*	MFLUCC 16–0021	MH558803	–	MH550996	MH551130
*Tubeufia sympodihylospora*	GZCC 16–0049	MH558804	MH558928	MH550997	MH551131
*Tubeufia sympodihylospora*	GZCC 16–0051	MH558805	MH558929	MH550998	MH551132
*Tubeufia sympodihylospora*	MFLUCC 17–0044	MH558806	MH558930	MH550999	MH551133
*Tubeufiasympodilaxispora* ^T^	MFLUCC 17–0048	MH558808	MH558932	MH551001	MH551135
*Tubeufia taiwanensis*	BCRC FU30844	LC316605	–	–	–
*Tubeufiatectonae* ^T^	MFLUCC 12–0392	KU144923	KU764706	KU872763	–
*Tubeufia tectonae*	MFLUCC 16–0235	MH558809	MH558933	MH551002	MH551136
*Tubeufia tectonae*	MFLUCC 15–0974	–	MN913688	MT954376	–
*Tubeufiatratensis* ^T^	MFLUCC 17–1993	MH558811	MH558935	MH551004	MH551138
*Tubeufia xylophila*	GZCC 16–0038	MH558812	MH558936	MH551005	MH551139
*Tubeufia xylophila*	MFLUCC 17–1520	MH558813	MH558937	MH551006	MH551140

Ex-type strains are indicated by ^T^ after the species name. Newly generated sequences are indicated in bold. The symbol “–” indicates information unavailable.

## Results

### Phylogenetic analyses

**Figure 1:**
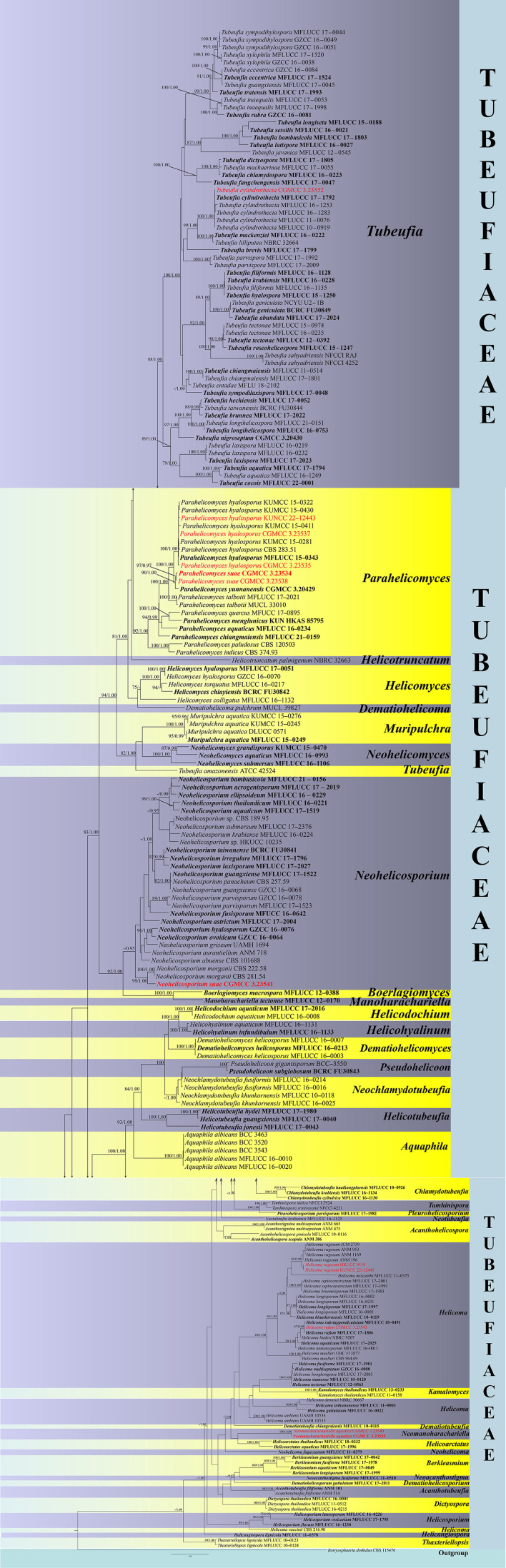
Phylogram generated from maximum likelihood analysis (RAxML) of *Tubeufiaceae* based on ITS, LSU, *tef 1-*α, and RPB2 sequence data. Maximum likelihood bootstrap values equal to or above 75% and Bayesian posterior probabilities (PP) equal to or above 0.95 are given above the nodes. The tree is rooted at *Botryosphaeria dothidea* CBS 115476. Newly-generated sequences are indicated in red. Ex-type strains are indicated in black/red bold.

Phylogenetic analyses of combined ITS, LSU, *tef 1-*α, and RPB2 sequences comprised a total of 3,316 characters including gaps, ITS (1–534 bp), LSU (535–1,362 bp), *tef 1-*α (1,363–2,273 bp), and RPB2 (2,274–3,316 bp) including 217 strains, with *Botryosphaeria dothidea* (CBS 115476) as the outgroup taxon. RAxML and Bayesian analyses of the combined dataset resulted in phylogenetic reconstructions with largely similar topologies. The result of ML analyses with a final likelihood value of −53,732.520635 is shown in Figure 1. Alignment exhibits 1,618 distinct alignment patterns; the proportion of gaps and completely undetermined characters in this alignment is 27.38%. Gamma distribution shape parameter: α = 0.226507; tree-length: 6.955943; estimated base frequencies: *A* = 0.242825, *C* = 0.253033, *G* = 0.260763, and *T* = 0.243379; substitution rates: AC = 1.238257, AG = 6.612700, AT = 2.116761, CG = 0.859127, CT = 10.120846, and GT = 1.000000. Bootstrap support values for RAxML >75% and Bayesian PP >0.95 are given at each node (Figure 1).

Phylogenetic analyses showed that the new isolates were nested in *Tubeufiaceae* with close affinities to four exciting genera, *viz*., *Helicoma, Neohelicosporium, Parahelicomyces, Tubeufia*, and the new genus *Neomanoharachariella*, forming a distinct clade among the genera of *Tubeufiaceae*. KUNCC 22–12445 and CGMCC 3.23543 clustered within *Helicoma*, sister to *Helicom rugosum* (ANM 196, ANM 953, ANM 1169, and JCM 2739) with 97% ML and 0.99 PP support values. Another strain, CGMCC 3.23543 nested in *H. rubriappendiculatum* (MFLUCC 18–0491) and *H. rufum* (MFLUCC 17–1806) with 87% ML and 0.99 PP support values. CGMC3.23541 nested in *N. morganii* (CBS 281.54) with strong bootstrap support (100% ML/1.00 PP). CGMC3.23539 and CGMCC 3.23540 clustered as a monophyletic clade sister to *Helicoarctatus aquaticus* (MFLUCC 17–1996) and *H. tailandicus* (MFLUCC 18–0332). Three new collections (CGMCC 3.23535, KUNCC 22–12443, and KUNCC 22–12444) clustered with *Parahelicomyces hyalosporus* (CBS 283.51, MFLUCC 15–0343, KUMCC 15–0430, KUMCC 15–0411, KUMCC 15–0322, and KUMCC 15–0281) with 100% ML and 1.00 PP support. CGMCC 3.23534 and CGMCC 3.23538 formed a sister lineage to *Parahelicomyces yunnanensis* (CGMCC 3.20429) with 90% ML and 1.00 PP support. CGMCC 3.23552 clustered with five strains of *Tubeufia cylindrothecia* (MFLUCC 10–0919, MFLUCC 11–0076, MFLUCC 16–1253, MFLUCC 16–1283, and MFLUCC 17–1792) with 100% ML and 1.00 PP support.

### Taxonomy

***Helicoma rugosum*** (C. Booth) Boonmee and K.D. Hyde [as 'rugosa'], Fungal Divers. 68: 266 (2014), Figure 2

**Figure 2:**
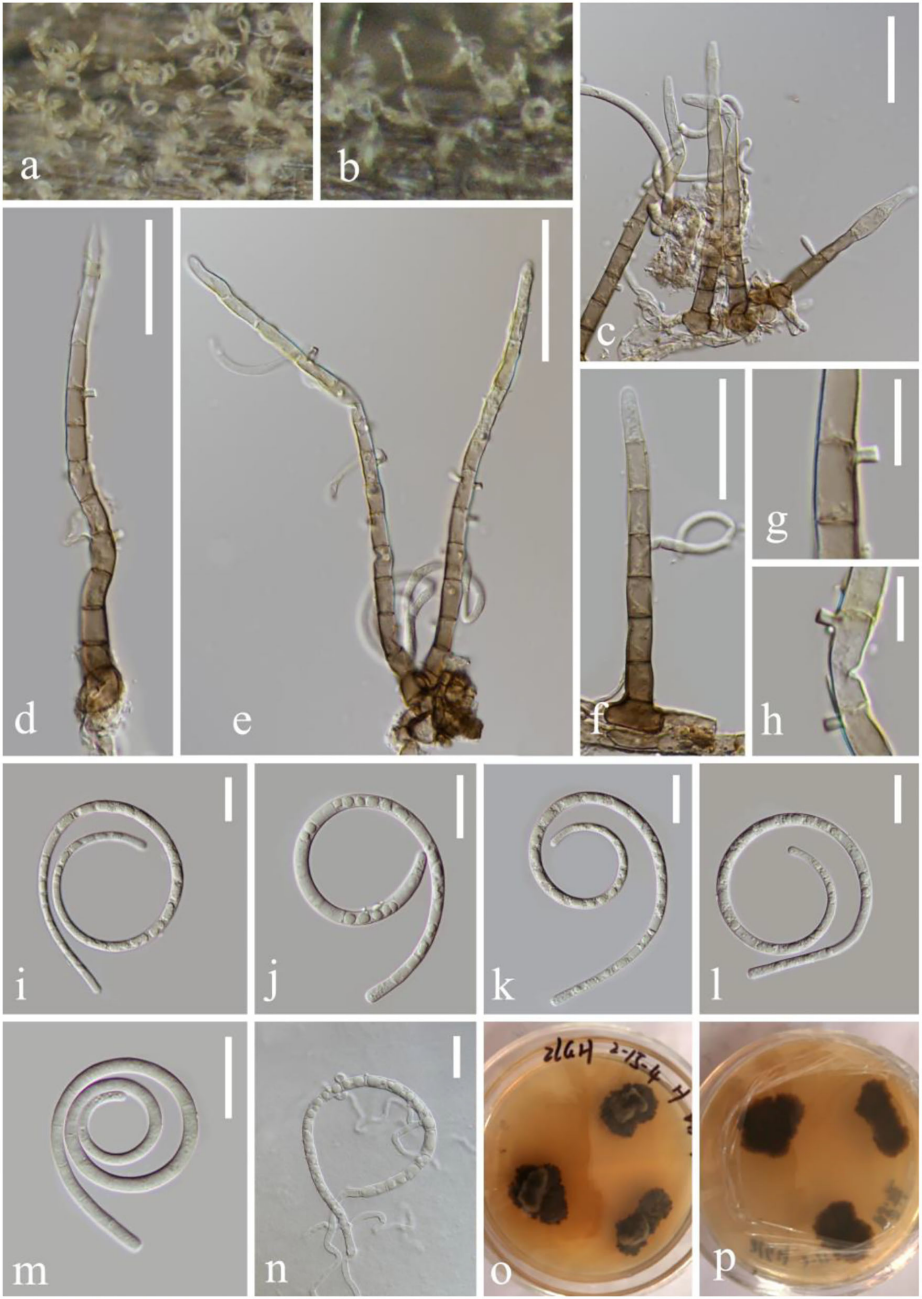
*Helicoma rugosum* (KUN-HKAS 124608). **(a,b)** Colony on decaying wood. **(c–f)** Conidiophores with attached conidia. **(g,h)** Conidiogenous cells. **(i–m)** Conidia. **(n)** Germinating conidium. **(o,p)** Colony on PDA observed from above and below. Scale bars: **(c,d)** 30 μm, **(e)** 50 μm, **(f)** 30 μm, **(g,h)** 10 μm, and **(i–n)** 20 μm.

*Index Fungorum*: IF 340543; *Facesoffungi number*: FoF 02650

*Saprobic* on submerged decaying wood in the lake. **Asexual morph:** Hyphomycetous, helicosporous. *Colonies* on natural substrate superficial, effuse, discrete, dilute, and light brown to brown. *Mycelium* composed of partly immersed, partly superficial, septate, pale brown to brown, branched hyphae, with masses of crowded, glistening conidia. *Conidiophores* 95–151 μm long, 5.4–6.8 μm wide ($\bar{\text{x}}$ = 122.6 × 6 μm, *n* = 20), macronematous, mononematous, straight to slightly bent, unbranched, septate, cylindrical, erect, pale brown to brown, and smooth-walled. *Conidiogenous cells* 9–12 μm long, 5–6 μm wide, holoblastic, mono- to polyblastic, integrated, intercalary, cylindrical, with denticles, tiny tooth-like protrusions (0.9–2.6 μm long, 0.5–1.7 μm wide), brown, and smooth-walled. *Conidia* 60.7–85.5 μm diameter, conidial filament 4–4.8 μm wide ($\bar{\text{x}}$ = 73 × 4.4 μm, *n* = 20), 216–290 μm long, slightly coiled 1.0–2.5 times, pleurogenous, helicoid, rounded at tip, septate, becoming loosely coiled in water, guttulate, pale brown, and smooth-walled. **Sexual morph:** not observed.

*Culture characteristics*: Conidia germinating on PDA and germ tubes produced from conidia within 12 h. Colonies growing on PDA, irregular, center umbonate, with a rough surface, wrinkle, edge undulate, reaching 10–15 mm in 2 weeks at 26°C, and pale brown to brown in the PDA medium. Mycelium superficial and partially immersed, branched, septate, hyaline to pale brown, and smooth-walled.

*Material examined*: China, Yunnan Province, Luguhu lake, on submerged decaying wood, 22 October 2021 (Altitude: 2,625 m, 27°42'41“N, 100°46'48“E), Long-Li Li, L-1013 (KUN-HKAS 124608), living culture, KUNCC 22–12445.

*Notes*: *Helicoma rugosum* was reported by Boonmee et al. (2014) to combine *Sphaeria helicoma, Thaxteriella helicoma*, and *Tubeufia rugosa* based on phylogenetic and morphological evidence. *H. rugosum* (KUNCC 22–12445) resembles *H. rufum*, presenting macronematous, mononematous, unbranched or branched, septate conidiophores, holoblastic, mono- to ployblastic conidiogenous cells, helicoid, and septate conidia. However, *H. rugosum* (KUNCC 22–12445) is distinct from *H. rufum* as it has shorter and narrower conidiophores (95–151 × 5.4–6.8 vs. 110–210 × 7–8.5 μm), longer and wider conidia (60.7–85.5 × 4–4.8 vs. 35–45 × 4–5.5 μm), and shorter conidial filaments (216–290 × 4–5 vs. 240–410 × 4–5.5 μm). Furthermore, *H. rufum* produces a reddish brown pigment in the PDA medium in 7 days but *H. rugosum* lacks this characteristic. In the phylogenetic analyses, *H. rugosum* (KUNCC 22–12445) cluster together with *H. rugosum* (ANM 196, ANM 1169, ANM 953, and JCM 2739) and *Helicoma* sp. (HKUCC 9118) with strong support (91% ML and 0.99 PP). In this study, we introduce our new collection with *Helicoma* sp. (HKUCC 9118) as *H. rugosum* because of identical LSU nucleotide sequences and morphological characteristics. Our fresh collection is morphologically similar to *Helicoma* sp. (HKUCC 9118) (Kodsueb et al., 2004) in terms of conidiogenous cells with tiny tooth-like protrusions, dentical, conidiophores brownish-gray, upright, and the same conidia size (61–86 × 4–5 vs. 37–86.4 × 4.6–5.4 μm). Furthermore, both of their morphologies fit into the generic group *Helicoma*, and the analyses show that they should be the same species.

***Helicoma rufum*** Y.Z. Lu, J.C. Kang, and K.D. Hyde, Fungal Divers. 92: 183 (2018), Figure 3

**Figure 3:**
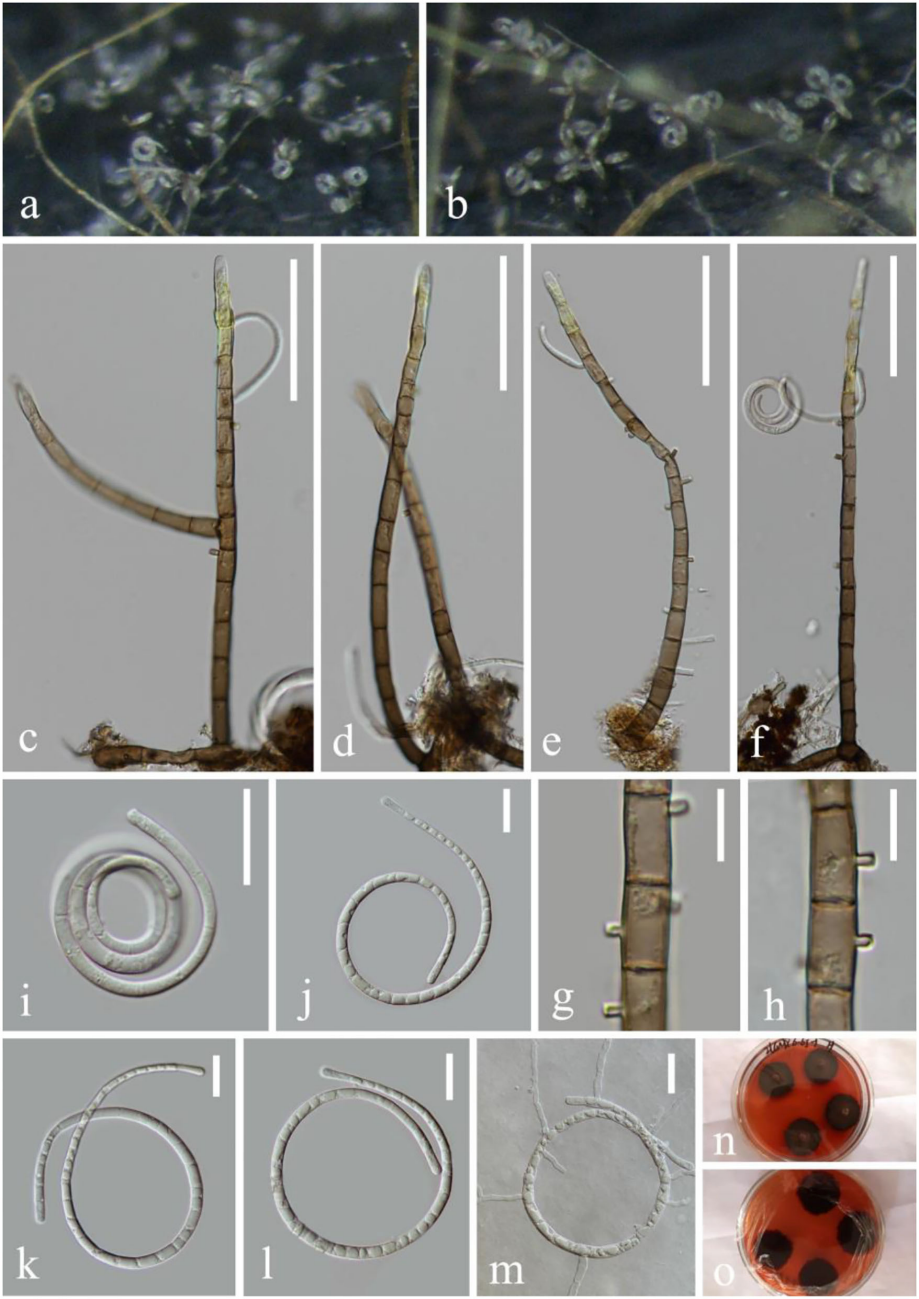
*Helicoma rufum* (KUN-HKAS 124609). **(a,b)** Colony rises from mycelium on natural wood substrate. **(c–f)** Conidiophores with attached conidia. **(g,h)** Conidiogenous cells. **(i–l)** Conidia. **(m)** Germinating conidium. **(n,o)** Culture on PDA. Scale bars: **(c–f)** 60 μm, **(g,h)** 10 μm, and **(i–m)** 20 μm.

*Index Fungorum*: IF 554843; *Facesoffungi number*: FoF 04718

*Saprobic* on submerged decaying wood in the lake. **Asexual morph**: Hyphomycetous, helicosporous. *Colonies* superficial, effuse, gregarious, and brown. *Mycelium* composed of immersed, partly superficial, hyaline to pale brown, septate, branched hyphae, with masses of crowded, glistening conidia. *Conidiophores* 136–209 μm long, 6–7 μm wide ($\bar{\text{x}}$ = 173 × 6.5 μm, *n* = 30), macronematous, mononematous, cylindrical, erect, straight to slightly bent, mostly unbranched, septate, the lower part brown and the upper part pale yellow, and smooth-walled. *Conidiogenous cells* 12–14 μm long, 5–7 μm wide, holoblastic, mono- to polyblastic, integrated, intercalary, cylindrical, with denticles, rising laterally from the lower portion of conidiophores as tiny tooth-like protrusions (2.7–3.9 μm long, 1.5–2.3 μm wide), brown, and smooth-walled. *Conidia* 57–104 μm diameter, conidial filament 3.4–5.2 μm wide ($\bar{\text{x}}$ = 80.6 × 4.3 μm, *n* = 20), 248–327 μm long, solitary, pleurogenous, helicoid, rounded at tip, septate, slightly constricted at septa, loosely coiled 1.5–3.5 times, becoming loosely coiled in water, guttulate, hyaline to pale brown, and smooth-walled. **Sexual morph**: not observed.

*Culture characteristics*: Conidia germinating on PDA within 12 h and many germ tubes produced from conidium cells. Colonies growing on PDA, reaching 25 mm, and started producing reddish brown pigment in 3 weeks at 26°C, brown to reddish brown in the PDA medium, irregular, with a flat surface, edge slightly undulate. Mycelium superficial and partially immersed, branched, septate, hyaline to pale brown, and smooth-walled.

*Material examined*: China, Yunnan Province, Luguhu lake, on submerged decaying wood (Altitude: 2,717 m, 27°42'41“N, 100°46'48“E), 21 October 2021, Long-Li Li, L-1032 (KUN-HKAS 124609), living cultures, CGMCC 3.23543 = KUNCC 22–12439.

*Notes*: *Helicoma rufum* was introduced by Lu et al. (2018b) on decaying wood in a mountain in Thailand. The new isolate L-1032 collected from freshwater habitats was identified as *H. rufum* based on the phylogenetic analyses and the morphological features. Our new collection CGMCC 3.23543 clusters in the same clade with *H. rufum* (MFLUCC 17–1806) and *H. rubriappendiculatum* (MFLUCC 18–0491) with bootstrap support (87% ML and 0.99 PP). Morphologically, our new collection is almost identical to *H. rufum* (MFLUCC 17–1806) except for the conidia diameter (57–104 vs. 35–45 μm long). The nucleotide comparisons show 4 bp, 1 bp, and 2 bp of ITS, LSU, and *tef 1-*α differences between the new isolate CGMCC 3.23543 and *H. rufum* (MFLUCC 17–1806). Between *H. rubriappendiculatum* (MFLUCC 18–0491) and *H. rufum* (CGMCC 3.23543), there are 4, 2, and 6 bp of ITS, LSU, and *tef 1-*α differences; compared with *H. rubriappendiculatum, H. rufum* (CGMCC 3.23543) produces a reddish brown pigment in the PDA medium and presents a longer conidia diameter (57–104 vs. 25–35 μm), lacking the characteristic red appendant near the apex in conidiophores. Thus, we identify the new isolate as *H. rufum* based on both phylogenetic analyses and morphological characteristics. This is the first report of *H. rufum* in freshwater habitats and its occurrence in China.

***Neohelicosporium suae*** L.L. Li, H.W. Shen and Z.L. Luo, **sp. nov**.

**Figure 4:**
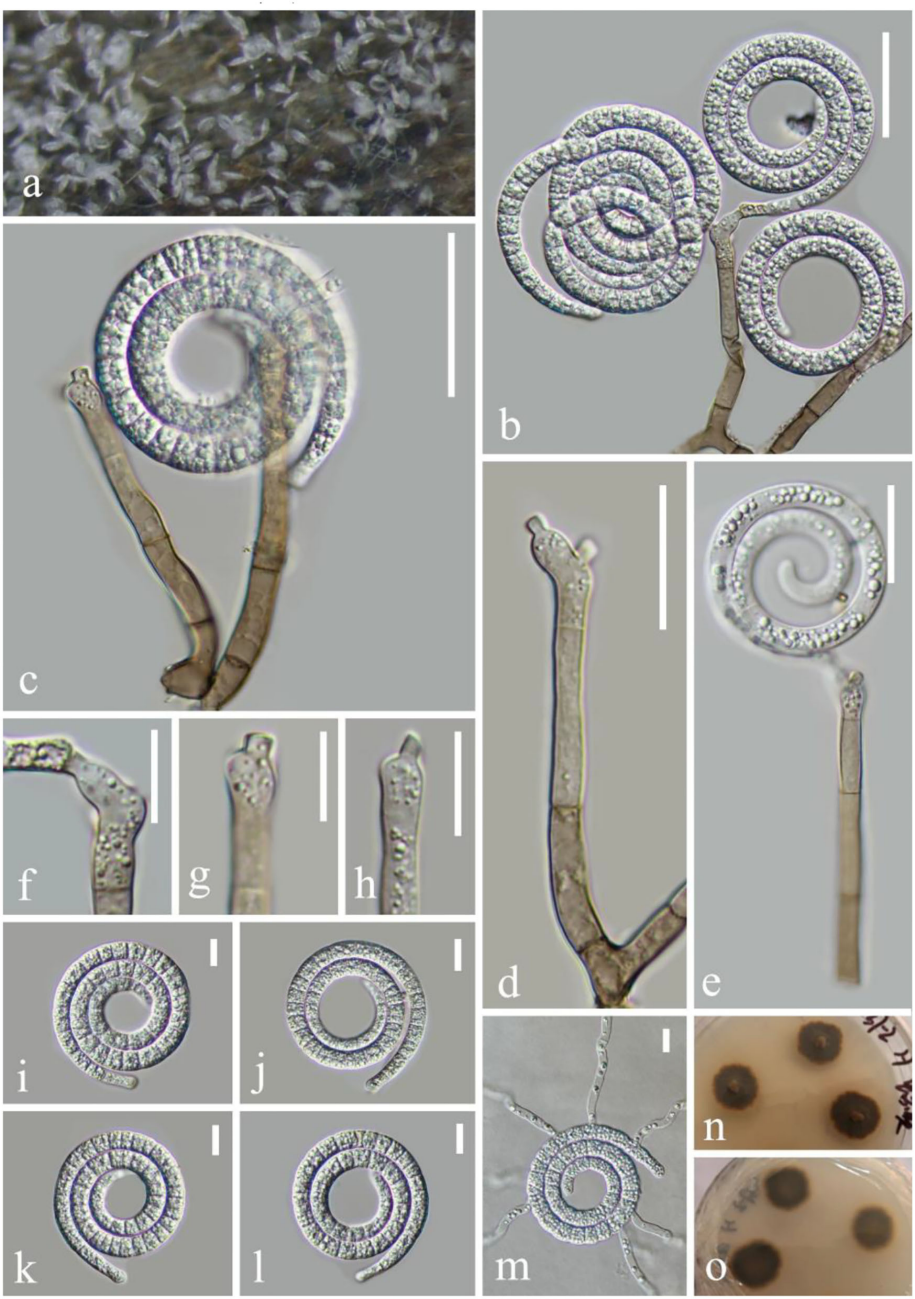
*Neohelicosporium suae* (KUN-HKAS 124610, holotype). **(a)** Colony on decaying wood. **(b,c,e)** Conidiophores with attached conidia. **(d)** Conidiophores. **(f–h)** Conidiogenous cells. **(i–l)** Conidia. **(m)** Germinating conidium. **(n,o)** Colony on PDA observed from above and below. Scale bars: **(b,c)** 30 μm, **(d,e)** 20 μm, and **(f–m)** 10 μm.

*MycoBank number*: MB 845321, Figure 4

Holotype—KUN-HKAS 124610

Etymology—“suae” (Lat.) in memory of the Chinese mycologist Prof. Hong-Yan Su (4 April 1967–3 May 2022).

*Saprobic* on submerged decaying wood in the lake. **Asexual morph**: Hyphomycetous, helicosporous. *Colonies* on substratum superficial, effuse, and white. *Mycelium* composed of superficial, partly immersed, brown, septate, branched hyphae, with crowded by conidial masses. *Conidiophores* 52–97 μm long, 4.2–5.1 μm wide ($\bar{\text{x}}$ = 75 × 4.7 μm, *n* = 20), macronematous, mononematous, erect, cylindrical, unbranched or less branched, 3–6-septate, hyaline to pale brown, and smooth-walled. *Conidiogenous cells* 15–27 μm long, 3.5–5 μm wide ($\bar{\text{x}}$ = 21 × 4.2 μm, *n* = 20), holoblastic, mono- to polyblastic, cylindrical, truncate at apex after conidial secession, integrated, sympodial, terminal, cylindrical, with denticles 2–3 × 1.5–2.4 μm, hyaline to pale brown, and smooth-walled. *Conidia* 45–55 μm diameter, conidial filaments 5–7 μm wide ($\bar{\text{x}}$ = 50 × 6 μm, *n* = 20), 212–268 μm long, tightly coiled 2–2.5 times, helicoid, rounded at tip, multi-septate, slightly constricted at septa, guttulate, hyaline, not becoming loose in water, and smooth-walled. **Sexual morph**: not observed.

*Culture characteristics*: Conidia germinating on PDA within 8 h. Colonies growing on PDA, circular, with a flat surface, edge entire, reaching 28 mm in 3 weeks at room temperature, pale brown to brown in the MEA medium. Mycelium superficial and partially immersed, branched, septate, hyaline to pale brown, and smooth-walled.

*Material examined*: China, Yunnan Province, Luguhu lake, on submerged decaying wood in the lake (Altitude: 2,242 m, 26°48'29“N, 100°43'4.8“E), 21 October 2021, Long-Li Li, L-1030 (KUN-HKAS 124610, **holotype**), ex-type cultures, CGMCC 3.23541 = KUNCC 22–12438.

*Notes*: *Neohelicosporium suae* is introduced as a new species based on morphological and phylogenetic evidence. In phylogeny, *N. suae* (CGMCC 3.23541) is a sister to *N. morganii* with strong bootstrap support (100% ML and 1.00 PP). Based on pairwise nucleotide comparisons, the new strain *N. suiae* (CGMCC3.23541) is different from *N. morganii* (CBS 281.54) in 9/532 bp (1.69%) of the ITS and 3/804 bp (0.37%) of the LSU. Morphologically, *N. suae* can be distinguished from *N. morganii*; the conidiophores of *N. suae* are unbranched or less branched, the latter are branched and shorter (52–97 μm long, 4.2–5.1 μm wide vs. up to 145 μm long, 5–7 μm wide) (Zhao et al., 2007), and the number of septa is more than 6. The conidiogenous cells of *N. suiae* are 15–27 μm long, swollen, with longer and wider denticles (2–3 × 1.5–2.4 vs. 1–2.5 × 0.5–1.5 μm), terminal, whereas *N. morganii* displays no swelling. Furthermore, *N. suiae* is distinct from *N. morganii*, presenting distinguished conidia characteristics in terms of a larger diameter (45–55 × 5–7 vs. 17–23 × 3–4 μm).

***Neomanoharachariella*** L.L. Li, H.W. Shen, and Z.L. Luo, **gen. nov**.

*Mycobank number*: MB 845535

Etymology—The generic epithet, neo (Lat., new), refers to the similarity to *Manoharachariella*.

*Saprobic* on decaying wood in the lake. **Asexual morph**: Hyphomycetous, dictyosporous. *Colonies* on the substratum superficial, effuse, and dark brown. *Conidiophores* macronematous, mononematous, erect, cylindrical, unbranched, straight or flexuous, paler, and smooth-walled. *Conidiogenous cells* monoblastic, integrated, terminal, cylindrical, subhyaline to pale brown, and smooth-walled. *Conidia* holoblastic smooth, shiny, simple, broadly oval to ellipsoid, muriform, tuberculous at the top, white and pale brown when immature, becoming dark to black when mature, and pale yellow at the basal cell and brown at other parts. **Sexual morph**: not observed.

Type species: *Neomanoharachariella aquatica* L.L. Li, H.W. Shen, and Z.L. Luo.

**Figure 5:**
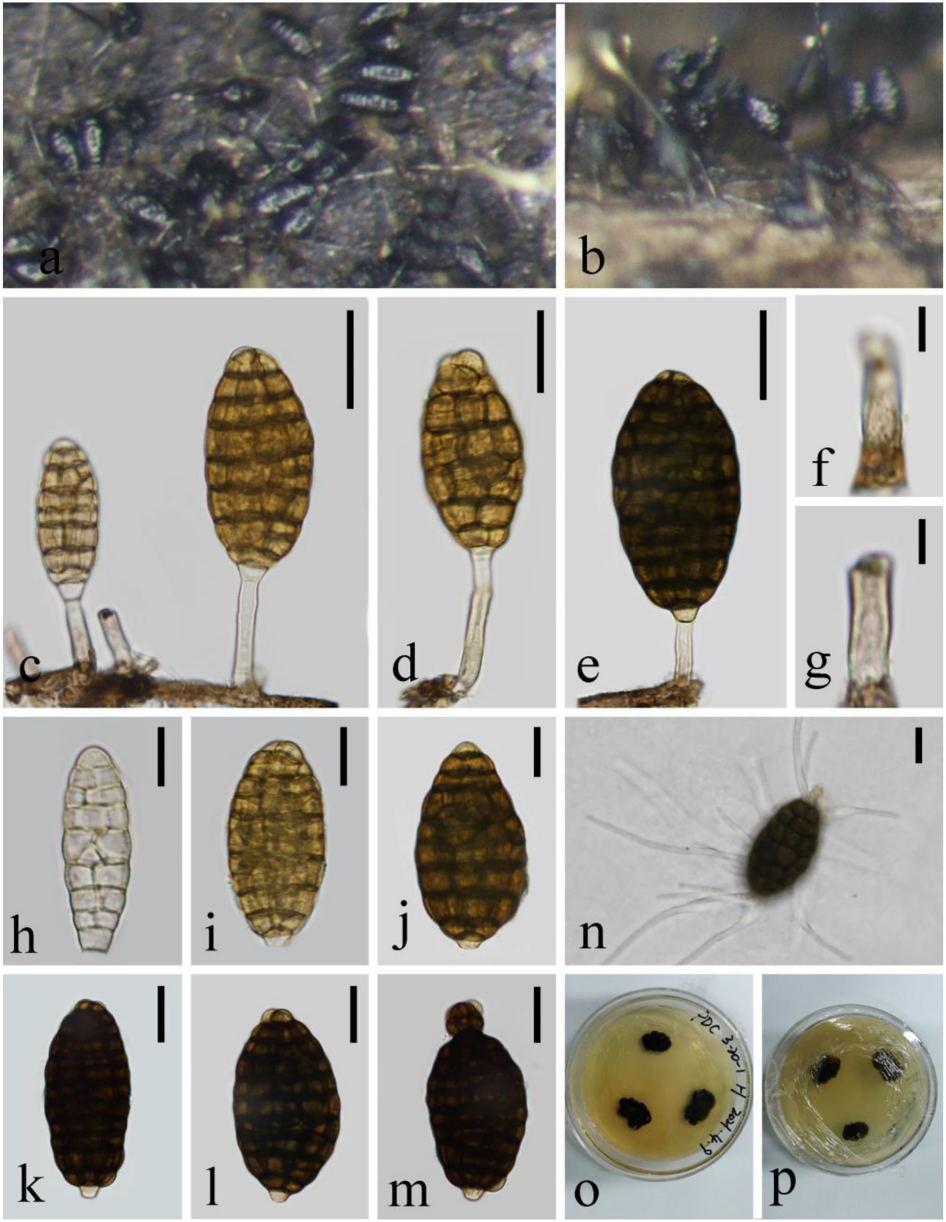
*Neomanoharachariella aquatica* (KUN-HKAS 124611, holotype). **(a,b)** Colony erect on decaying wood. **(c–e)** Conidiophores with attached conidia. **(f,g)** Conidiogenous cells. **(h–m)** Conidia. **(n)** Germinating conidium. **(o,p)** Culture on PDA. Scale bars: **(c,e)** 25 μm, **(f,g)** 5 μm, **(h–j)** 15 μm, and **(d,k–n)** 20 μm.

*Notes*: *Neomanoharachariella* is morphologically similar to *Chlamydotubeufia, Dictyospora*, and *Neochlamydotubeufia*, presenting dictyoseptate, broadly oval to ellipsoid, and darkened to black when matured conidia. However, *Neomanoharachariella* can be distinguished from other chlamydosporous genera by well-developed conidiophores. The morphological characteristics allow the assignment of *Neomanoharachariella* to *Tubeufiaceae*. In phylogeny, it formed a well-separated clade from all other genera of *Tubeufiaceae* (Figure 5). The molecular phylogenetic studies indicate its placement in *Tubeufiaceae* as a genus that is phylogenetically close to the genera, *Berkleasium, Dictyospora, Helicoarctatus, Helicoma*, and *Helicosporium*.

***Neomanoharachariella aquatica*** L.L. Li, H.W. Shen, and Z.L. Luo, **sp. nov**.

*Mycobank number*: MB 845536, Figure 5

Holotype—KUN-HKAS 124611

Etymology—“aquatica” referring to the aquatic habitat of this fungus.

*Saprobic* on decaying woods in the lake. **Asexual morph**: hyphomycetous, dictyosporous. *Colonies* on the substratum superficial, effuse, and dark brown. *Conidiophores* 20–31 μm long, 3.5–4.2 μm wide ($\bar{\text{x}}$ = 25 × 4 μm, *n* = 20), macronematous, mononematous, erect, cylindrical, unbranched, straight or flexuous, paler, and smooth-walled. *Conidiogenous cells* monoblastic, integrated, terminal, cylindrical, subhyaline to pale brown, and smooth-walled. *Conidia* 37–61 μm long, 17–32 μm wide ($\bar{\text{x}}$ = 49 × 24 μm, *n* = 20), muriform 8–10-transversely septate, with 1–4-longitudinal septa, smooth, shiny, simple, broadly oval to ellipsoid, tuberculous at the top, hyaline to pale brown when immature, becoming dark to black when mature, and pale yellow at the basal cells and brown at other parts. **Sexual morph**: not observed.

*Culture characteristics*: Conidia germinating on PDA within 12 h. Colonies growing on PDA, circular, with a flat surface, edge entire, reaching 15 mm in 3 weeks at 26°C, and brown to dark brown in the PDA medium. Mycelium superficial and partially immersed, branched, septate, hyaline to pale brown, and smooth-walled.

*Material examined*: China, Yunnan Province, Shuduhu lake, on submerged decaying wood (Altitude: 3,578 m, 27°54'24“N, 99°57'15“E), 25 August 2020, Zheng-Quan Zhang, L-190 (KUN-HKAS 124611, **holotype**), ex-type cultures, CGMCC 3.23539 = KUNCC 22–12437; China, Yunnan Province, Shuduhu lake, on submerged decaying wood (Altitude: 3,578 m, 27°54'24“N, 99°57'15“E), 25 August 2020, Zheng-Quan Zhang, L-281 (KUN-HKAS 124612), living cultures, CGMCC 3.23540 = KUNCC 22–12442.

*Notes*: The new collection can be easily distinguished from other *Tubeufiaceae* genera by the long oval and dictyosporous conidia with well-developed conidiophores. In the phylogenetic analyses, *Neomanoharachariella aquatica* shares a sister relationship to *Helicoarctatus aquaticus* (MFLUCC 17–1996) and *H. thailandicus* (MFLUCC 18–0332). However, there are great differences in morphology; the asexual morph of *H. aquaticus* and *H. thailandicus* are helicosporous, and our new collection is dictyosporous. *H. aquaticus* and *H. thailandicus* are characterized by setiform, unbranched, septate conidiophores, holoblastic, mono- to poly-blastic, denticulate conidiogenous cells, pleurogenous, helicoid, multi-septate, guttulate, and hyaline conidia. Based on pairwise nucleotide comparisons, the new strain CGMCC 3.23540 is different from the type species *Helicoarctatus aquaticu*s (MFLUCC 17–1996) in 30/541 bp (5.54%) of the ITS, 24/805 bp (2.98%) of the LSU, 74/875 bp (8.46%) of the *tef 1-*α, and 154/1045 bp (14.74%) of the RPB2. In addition, *Neomanoharachariella aquatica* is most similar to the asexual state of *Chlamydotubeufia huaikangplaensis*, but the conidia of *N. aquatica* are shorter (37–61 × 17–32 vs. 50–77 × 39–42) and presenting erect, unbranched, and smooth-walled conidiophores; the phylogenetic analyses also clearly segregate it from *C. huaikangplaensis*. We therefore identify the newly obtained taxon as *Neomanoharachariella aquatica* sp. nov.

***Parahelicomyces hyalosporus*** (Y.Z. Lu, J.K. Liu, and K.D. Hyde) S. Y. Hsieh, Goh, and C. H. Kuo, Mycol. Prog. 20(2): 182 (2021) Figure 6

**Figure 6:**
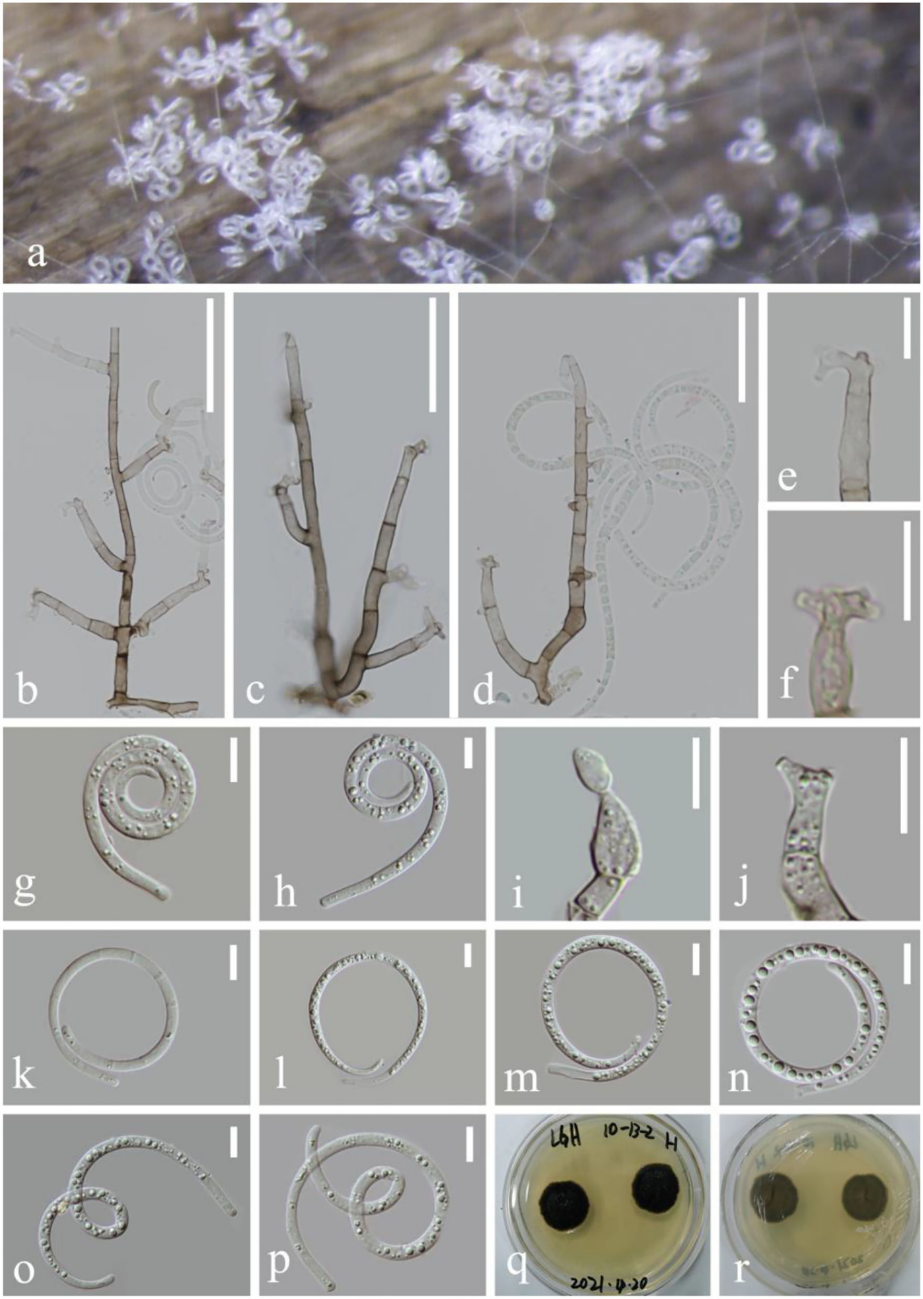
*Parahelicomyces hyalosporus* (KUN-HKAS 124603). **(a)** Colony on decaying wood. **(b–d)** Conidiophores with attached conidia and lateral minute polyblastic denticles. **(e,f,i,j)** Conidiogenous cells. **(g,h,k–p)** Conidia. **(p,q)** Colony on PDA observed from above and below. Scale bars: **(b)** 50 μm, **(c,d)** 40 μm, and **(e–p)** 10 μm.

*Index Fungorum*: IF 554888; *Facesoffungi number*: FoF 04812

*Saprobic* on submerged decaying woods in the lake. **Asexual morph**: Hyphomycetous, helicosporous. *Colonies* on wood substrate superficial, effuse, gregarious, and hyaline to white. *Mycelium* composed of partly immersed, partly superficial, pale brown, septate, anastomosing, reapent, with masses of crowded conidia. *Conidiophores* 60–142 μm long, 4–5.2 μm wide ($\bar{\text{x}}$ = 101 × 4.6 μm, *n* = 10), macronematous, mononematous, cylindrical, branched, septate, hyaline to pale brown, and smooth-walled. *Conidiogenous cells* 5–10 μm long, 3–4 μm wide, holoblastic, mono-to polyblastic, integrated, terminal or intercalary, cylindrical, truncate at apex after conidial secession, hyaline to pale brown, and smooth-walled. *Conidia* 40–56.7 μm diameter, and conidial filaments 3.5–4.5 μm wide ($\bar{\text{x}}$ = 48 × 4 μm, *n* = 20), 145–180 μm long, loosely coiled 1–2.5 times, solitary, pleurogenous or acropleurogenous, helicoid, rounded at tip, multi-septate, becoming loosely coiled in water, guttulate, hyaline, and smooth-walled. **Sexual morph**: not observed.

*Culture characteristics*: Conidia germinating on PDA within 12 h; many germ tubes produced from conidium cells. Colonies growing on PDA, circular, with umbonate surface, edge dulate, and brown to dark brown in PDA medium, reaching 20 mm in 3 weeks at 26°C, and brown to dark brown in the PDA medium. Mycelium superficial and partially immersed, branched, septate, hyaline to pale brown, and smooth-walled.

*Material examined*: China, Yunnan Province, Luguhu lake, on submerged decaying wood (Altitude: 2,698 m, 27°41'11“N, 100°48'18“E), 5 March 2021, Zheng-Quan Zhang, L-159 (KUN-HKAS 124603), living cultures, CGMCC 3.23535 = KUNCC 22–12436; China, Yunnan Province, Luguhu lake, on submerged decaying wood (Altitude: 2734 m, 27°45'18“N, 100°46'42“E), 5 March 2021, Zheng-Quan Zhang, L-315 (KUN-HKAS 124606), living culture, KUNCC 22–12443; China, Yunnan Province, Luguhu lake, on submerged decaying wood (Altitude: 2,794 m, 27°45'02“N, 100°51'02“E), 5 March 2021, Zheng-Quan Zhang, L-326 (KUN-HKAS 124605), living cultures, CGMCC 3.23537 = KUNCC 22–12444.

*Notes*: *Parahelicomyces hyalosporus* was first introduced as *Pseudohelicomyces hyalosporus* by Lu et al. (2018b) based on morphological and phylogenetic evidence. Hsieh et al. (2021) transferred it to *Parahelicomyces* as the genus *Pseudohelicomyces* was an older homonym and illegitimate. In this paper, three newly-obtained isolates were identified as *Parahelicomyces hyalosporus*, and the morphology characteristics fit well with *Parahelicomyces hyalosporus*; the conidiophores macronematous, mononematous, branched, septate, conidiogenous cells with denticles, holoblastic, mono- to polyblastic, intercalary or terminal, determinate or sympodial and pleurogenous or acropleurogenous, conidia helicoid, multi-septate, and hyaline to pale brown. Species of the *P. hyalosporus* are widely found in lakes and streams of freshwater habitats in China and Thailand (Luo et al., 2017; Lu et al., 2018b; Li et al., 2022). Based on pairwise nucleotide comparisons, ITS and LSU are identical between the type species (MFLUCC 15–0343) and *P. hyalosporus* (CGMCC 3.23535).

***Parahelicomyces suae*** L.L. Li, H.W. Shen, and Z.L. Luo, **sp. nov**.

**Figure 7:**
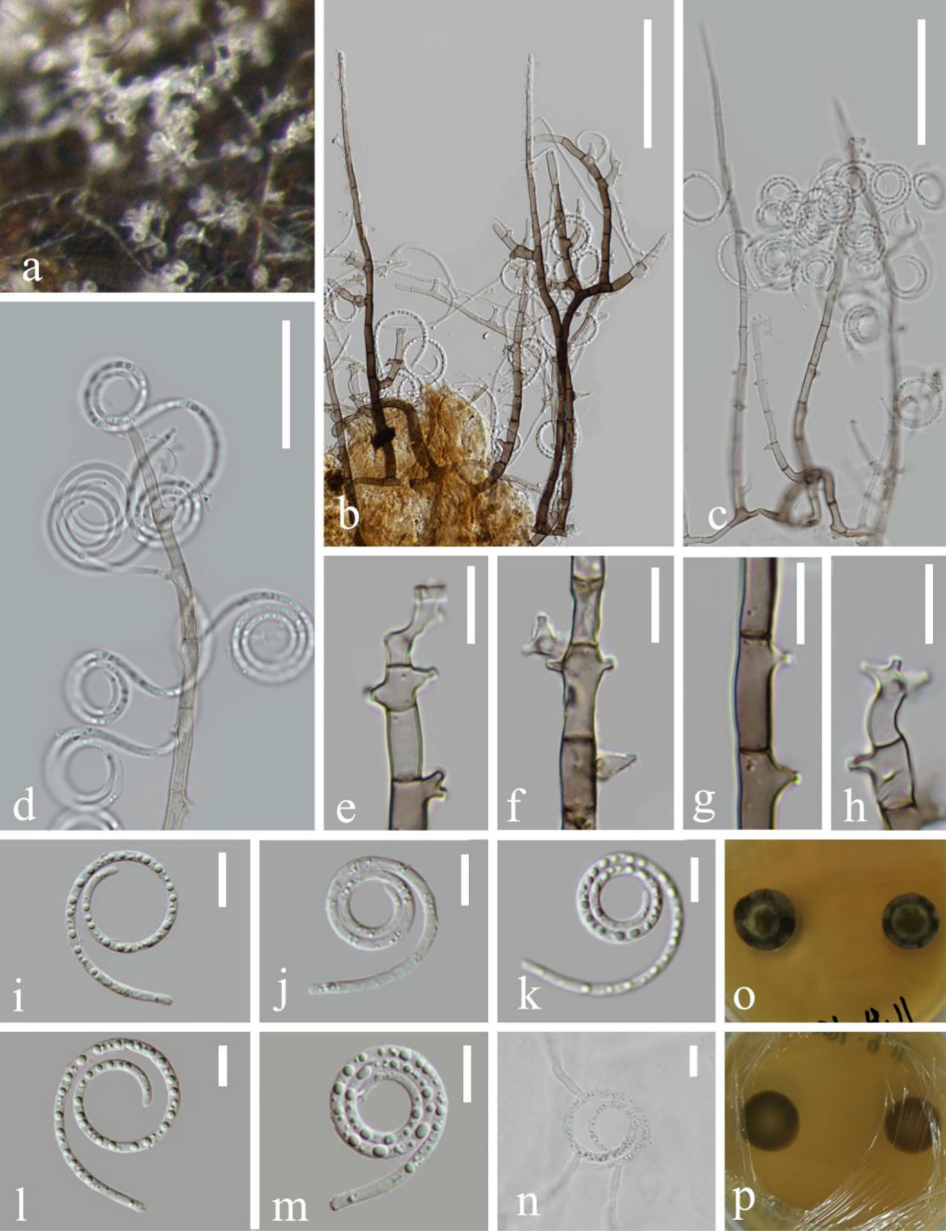
*Parahelicomyces suae* (KUN-HKAS 124604, holotype). **(a)** Colony on decaying wood. **(b–d)** Conidiophores with attached conidia. **(e–h)** Conidiogenous cells. **(i–m)** Conidia. **(n)** Germinating conidium. **(o,p)** Colony on MEA observed from above and below. Scale bars: **(b)** 70 μm, **(c)** 60 μm, **(d)** 30 μm, **(e–h,j–n)** 10 μm, and **(i)** 15 μm.

*Mycobank number*: MB 845534, Figure 7

Holotype—KUN-HKAS 124604

Etymology—“suae” (Lat.) in memory of the Chinese mycologist Prof. Hong-Yan Su (4 April 1967–3 May 2022).

*Saprobic* on submerged decaying woods in the lake. **Asexual morph**: Hyphomycetous, helicosporous. *Colonies* on the wood substratum superficial, effuse, gregarious, and white. *Mycelium* composed of partly immersed, partly superficial, hyaline to pale brown, septate, abundantly branched hyphae, with masses of crowded, glistening conidia. *Conidiophores* 114.8–173.5 μm long, 3–4 μm wide ($\bar{\text{x}}$ = 144 × 3.5 μm, *n* = 20), macronematous, mononematous, cylindrical, branched or unbranched, erect, septate, dark brown at base, becoming hyaline toward apex, and smooth-walled. *Conidiogenous cells* 12–18 μm long, 3–4 μm wide, sympodial, holoblastic, monoblastic, integrated, terminal, cylindrical, truncate at apex after conidial secession, denticles or bladder-like cells, hyaline to pale brown, and smooth-walled. *Conidia* 29–36 μm diameter, conidial filament 1.8–2.2 μm wide ($\bar{\text{x}}$ = 32.5 × 2 μm, *n* = 20), 103–121 μm long, coiled 1–3.5 times, solitary, helicoid, rounded at tip, young conidia have indistinct septate, not easily loosely coiled in water, guttulate, hyaline, and smooth-walled. **Sexual morph**: not observed.

*Culture characteristics*: Conidia germinating on PDA within 12 h and many germ tubes produced from conidium cells. Colonies growing on MEA, reaching 14 mm diameter in 2 weeks at 26°C, circular, with a flat surface, edge entire, and pale brown to brown in the MEA medium. Mycelium superficial and partially immersed, branched, septate, hyaline to pale brown, and smooth.

*Material examined*: China, Yunnan Province, Luguhu lake, on submerged decaying wood in the lake (Altitude: 2,698 m, 27°41'11“N, 100°48'18“E), 3 March 2021, Sha Luan, L-158 (KUN-HKAS 124604, **holotype**), ex-type cultures, CGMCC 3.23534 = KUNCC 22–12435; China, Yunnan Province, Luguhu lake, on submerged decaying wood in the lake (Altitude: 2698 m, 27°42'43“N, 100°44'56“E), 3 March 2021, Long-Li Li, L-1038, (KUN-HKAS 124607), living cultures, CGMCC 3.23538 = KUNCC 22–12440.

Notes: *Parahelicomyces suae* is introduced as a new species from Luguhu lake in Yunnan, China. In phylogeny, *P. suae* constitutes a strongly supported independent lineage basal to *P. yunnanensis*. Compared with CGMCC 3.20429, there are 5/563 (0.89%), 11/1048 bp (1.05%) base pair differences in the ITS and RPB2 regions between these two species. Morphologically, compared with *P. yunnanensis*, the conidia of *P. suae* are shorter (103–121 vs. 104–156 μm). In addition, our isolate conidia are not easily loosely coiled in water, conidiogenous cells with denticulate, and hyaline. Therefore, we identify the isolate as a new species of *P. suae*.

***Tubeufia cylindrothecia*** (Seaver) Höhn Sber. Akad. Wiss. Wien, Math.-naturw. Kl., Abt. 1 128: 562 (1919), Figure 8

**Figure 8:**
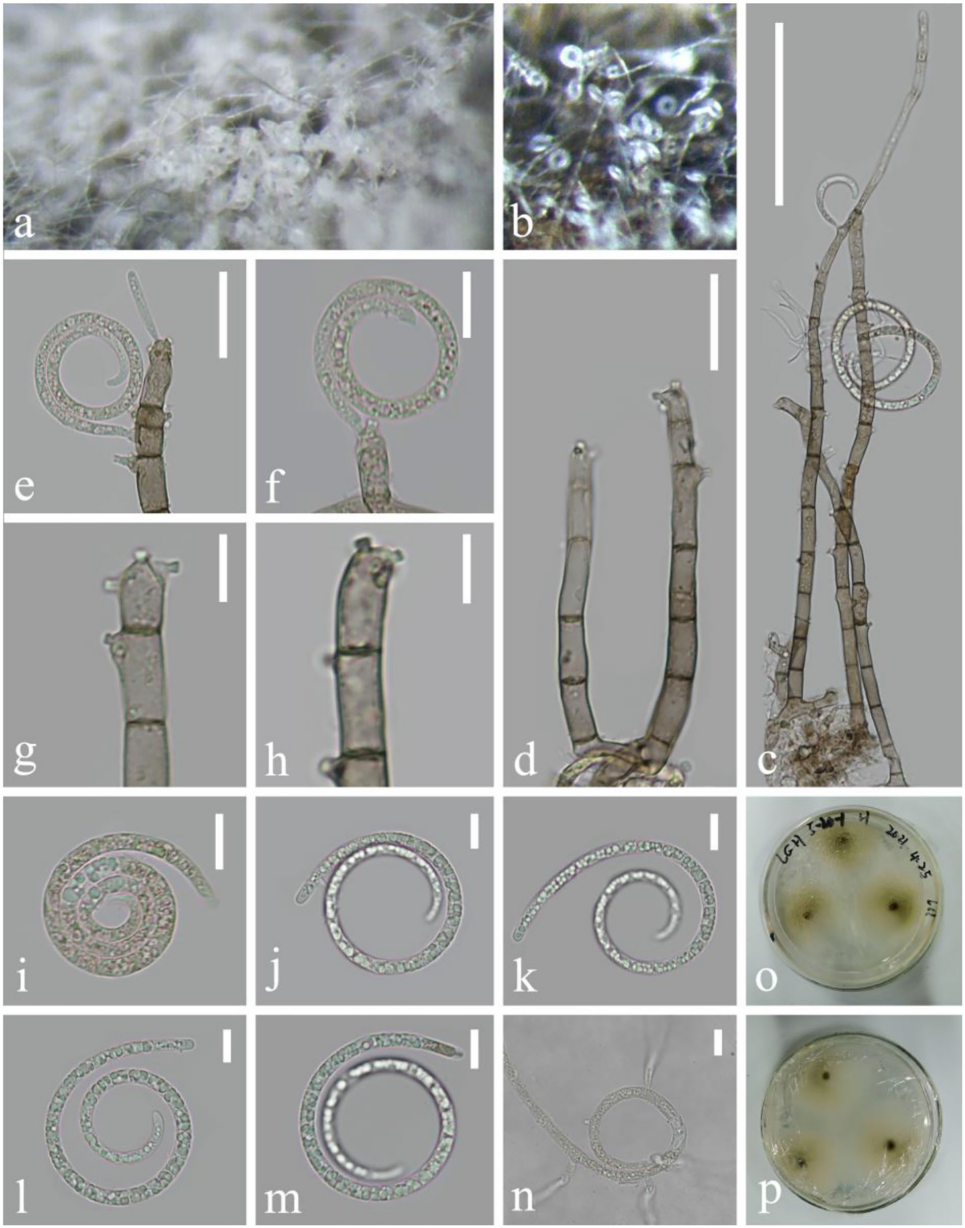
*Tubeufia cylindrothecia* (KUN-HKAS 124602). **(a,b)** Colony on decaying wood. **(c)** Conidiophores with attached conidia. **(d)** Conidiophores. **(e–h)** Conidiogenous cells. **(i–m)** Conidia. **(n)** Germinating conidium. **(o,p)** Colony on CMA observed from above and below. Scale bars: **(c)** 70 μm, **(d,e)** 20 μm, and **(f–n)** 10 μm.

*Index Fungorum*: IF 340543; *Facesoffungi number*: FoF 02650

*Saprobic* on decaying wood in the lake. **Asexual morph**: Hyphomycetous, helicosporous. *Colonies* on the substratum superficial, effuse, gregarious, and white to pale brown. *Mycelium* composed of partly immersed, partly superficial, hyaline to pale brown, septate, abundantly branched hyphae, with masses of crowded, glistening conidia. *Conidiophores* 97–200 μm long, 5–6 μm wide ($\bar{\text{x}}$ = 148 × 5.5 μm, *n* = 30), macronematous, mononematous, cylindrical, branched or unbranched, erect, flexuous, pale brown to brown, and smooth-walled. *Conidiogenous cells* 10.4–17 × 4–6 μm ($\bar{\text{x}}$ = 13.7 × 5 μm, *n* = 30), holoblastic, mono- to polyblastic, integrated, intercalary or terminal, cylindrical, repeatedly geniculate, truncate at the apex after conidial secession, each with single or several conidia hyaline to pale brown, and smooth-walled. *Conidia* 41.6–57.8 μm diameter and conidial filament 3.7–4.9 μm wide ($\bar{\text{x}}$ = 50 × 4.3 μm, *n* = 30), 105–206 μm long, coiled 1.5–3.5 times, solitary, acrogenous or acropleurogenous, helicoid, rounded at tip, becoming loosely coiled in water, guttulate, young Conidia hyaline and pale brown when edged, and smooth-walled. **Sexual morph**: not observed.

*Culture characteristics*: Conidia germinating on PDA within 12 h. Colonies growing slowly on CMA, reaching 15 mm diameter after 2 weeks at 26°C, effuse, the middle is dark, velvety to hairy, edge undulate, brown to dark brown in the CMA medium, mycelium superficial, effuse, with irregular edge, and hyphae pale yellow to brown.

*Material examined*: China, Yunnan Province, Luguhu lake, on submerged decaying wood (Altitude: 2,734 m, 27°45'18“N, 100°46'42“E), 5 March 2021, Zheng-Quan Zhang, L-157 (KUN-HKAS 124602), living cultures, CGMCC 3.23552 = KUNCC 22–12434.

*Notes*: The asexual morph of *Tubeufa cylindrothecia* was first reported by Luo et al. (2017) and later encountered by Lu et al. (2018b) in freshwater habitats. In this study, the newly obtained collection has longer conidiophores (97–200 vs. 50–81 μm) and shorter conidia (105–206 vs. 256–314 μm) compared with the holotype (Luo et al., 2017). However, their ITS, LSU, *tef 1-*α, and RPB2 sequence data are identical; we therefore identify it as *Tubeufia cylindrothecia*.

## Discussion

The modern classification of *Tubeufiaceae* was established by Boonmee et al. (2014), based on phylogenetic analyses and morphology. However, there are still taxonomic confusions in this group, especially in those types with helicosporous asexual morphs; their morphologically-based intergeneric classifications are controversial. Some species have been transferred or are synonymous to other genera of *Tubeufiaceae*, for example, *Helicosporium pannosum, Neohelicosporium griseum*, and *N. morganii* have been transferred several times. The asexual state of *Neomanoharachariella* is dictyosporous conidia. It is a unique tubeufiaceous fungus with broadly oblong, elongate, multiseptate, muriform conidia, at first pale brown, becoming dark brown, with well-developed conidiophores, and basal cells are hyaline and bulging. These characteristics make it distinct from all related *Tubeufiacceae* genera and is hence proposed as a new genus. Phylogenetic analyses based on ITS, LSU, *tef 1-*α, and RPB2 sequence (Figure 1) also distinguish *N. aquatica* from other dictyosporous members of *Tubeufiaceae*. The new genus is related to *Helicoarctatus aquaticus* (MFLUCC 17–1996) and *Helicoarctatus thailandicus* (MFLUCC 18–0332) which formed a distinct clade. The phylogenetic analyses also clearly segregated other dictyosporous genera of *Tubeufiaceae* such as *Chlamydotubeufia, Dictyospora, Manoharachariella*, and *Tamhinispora* in well-differentiated monophyletic lineages.

An abundance of lakes is a major feature of the Yunnan plateau. In recent years, lignicolous freshwater fungi were investigated in Yunnan, in nine freshwater lakes on the plateau. These lakes are distributed in high-altitude areas and most of them are depression pools formed by the subsidence of faults, with no water channels connected (Yang et al., 2004; Shen et al., 2022). Because of their unique development, formation, and relativele isolation, each lake possesses its own unique species. In this study, we have also examined seven tubuefiaceous species collected from these plateau lakes. Of which, three were introduced as new species and a new genus *Neomanoharachariella*, while four were identified as existing species based on phylogenetic analyses and morphological characteristics. The nine species were placed in *Helicoma, Neohelicosporium, Parahelicomyces*, and *Tubeufia*. This study provides a case study for lakes as a worthwhile niche area of hyphomycetous associations. *Parahelicomyces* is well studied, and eight species in this genus have sequence data in the GenBank. For the common and confusing genera *Helicoma, Neohelicosporium*, and *Tubeufia*, morphological characteristics (conidiophores, conidiogenous cells, and conidia including size and color) and phylogenetic analyses are essential to distinguish them.

In conclusion, some tubeufiaceous species have the potential to produce new structural and active secondary metabolites (Mao et al., 2014; Lu et al., 2018a). Fang et al. (2019) tested and reported that most *Tubeufiaceae* species have certain antibacterial and anti-tumor activities *in vitro*. At present, few studies have reported secondary degradation products of *Helicoma, Helicomyces*, and *Helicosporium* species. In view of the potential to produce active compounds, and the reports on secondary metabolites of *Tubeufiaceae*, the prospect of active research is broad, and it is very possible to obtain new compounds with various biological activities from *Tubeufiaceae*.

## Data availability statement

The datasets presented in this study can be found in online repositories. The names of the repository/repositories and accession number(s) can be found in the article/supplementary material.

## Author contributions

L-LL conducted the experiments, analyzed the data, and wrote the manuscript. D-FB, DW, and Y-ZL revised the manuscript. H-WS planned the experiments and analyzed the data. Z-LL planned and funded the experiments. YF conducted the experiments. All authors contributed to the article and approved the submitted version.

## Funding

This work was mainly supported by the National Natural Science Foundation of China (Project ID: 32060005 and 31900020) and the Yunnan Fundamental Research Project (Grant Nos. 202101AU070137 and 202201AW070001).

## Conflict of interest

The authors declare that the research was conducted in the absence of any commercial or financial relationships that could be construed as a potential conflict of interest.

## Publisher's note

All claims expressed in this article are solely those of the authors and do not necessarily represent those of their affiliated organizations, or those of the publisher, the editors and the reviewers. Any product that may be evaluated in this article, or claim that may be made by its manufacturer, is not guaranteed or endorsed by the publisher.
